# Biogenesis of flavor-related linalool is diverged and genetically conserved in tree peony (*Paeonia* × *suffruticosa*)

**DOI:** 10.1093/hr/uhac253

**Published:** 2022-11-07

**Authors:** Shanshan Li, Ling Zhang, Miao Sun, Mengwen Lv, Yong Yang, Wenzhong Xu, Liangsheng Wang

**Affiliations:** Key Laboratory of Plant Resources, Institute of Botany, Chinese Academy of Sciences, Beijing 100093, China; China National Botanical Garden, Beijing 100093, China; University of Chinese Academy of Sciences, Beijing 100049, China; Key Laboratory of Plant Resources, Institute of Botany, Chinese Academy of Sciences, Beijing 100093, China; China National Botanical Garden, Beijing 100093, China; University of Chinese Academy of Sciences, Beijing 100049, China; Key Laboratory of Plant Resources, Institute of Botany, Chinese Academy of Sciences, Beijing 100093, China; School of Landscape Architecture, Beijing Forestry University, Beijing 100083, China; Key Laboratory of Plant Resources, Institute of Botany, Chinese Academy of Sciences, Beijing 100093, China; School of Landscape Architecture, Beijing Forestry University, Beijing 100083, China; Key Laboratory of Plant Resources, Institute of Botany, Chinese Academy of Sciences, Beijing 100093, China; China National Botanical Garden, Beijing 100093, China; Key Laboratory of Plant Resources, Institute of Botany, Chinese Academy of Sciences, Beijing 100093, China; China National Botanical Garden, Beijing 100093, China; University of Chinese Academy of Sciences, Beijing 100049, China; Key Laboratory of Plant Resources, Institute of Botany, Chinese Academy of Sciences, Beijing 100093, China; China National Botanical Garden, Beijing 100093, China; University of Chinese Academy of Sciences, Beijing 100049, China

## Abstract

Floral scent is an important and genetically complex trait in horticultural plants. Tree peony (*Paeonia* × *suffruticosa*) originates in the Pan-Himalaya and has nine wild species divided into two subsections, *Delavayanae* and *Vaginatae*. Their flowers are beloved worldwide for their sweet floral fragrance, yet the flavor-related volatiles and underlying biosynthetic pathways remain unknown. Here, we characterized the volatile blends of all wild tree peony species and found that the flavor-related volatiles were highly divergent, but linalool was a unique monoterpene in subsect. *Delavayanae*. Further detection of volatiles in 97 cultivars with various genetic backgrounds showed that linalool was also the characteristic aroma component in *Paeonia delavayi* hybrid progenies, suggesting that linalool was conserved and dominant within subsect. *Delavayanae* and its hybrids, instead of species and cultivars from subsect. *Vaginatae*. Global transcriptome analysis of all wild tree peony species and 60 cultivars revealed five candidate genes that may be involved in key steps of linalool biosynthesis; especially the expressions of three *TPS* genes, *PdTPS1*, *PdTPS2*, and *PdTPS4*, were significantly positively correlated with linalool emissions across tree peony cultivars. Further biochemical evidence demonstrated that *PdTPS1* and *PdTPS4* were the pivotal genes determining the species-specific and cultivar-specific emission of linalool. This study revealed a new insight into floral scent divergence in tree peony and would greatly facilitate our understanding of the phylogeny and evolution of *Paeonia*.

## Introduction

Floral scent is one of the strategies that flowering plants have evolved to adapt to surroundings, and the compounds responsible for it are widely used in cosmetics, flavorings, and the pharmaceutical industry [1, 2]. Furthermore, floral scent is one of the most important horticultural traits, yet is disadvantaged by over-focusing on visual attributes [3]. Recently, the biosynthesis of volatile compounds and their ecological roles have begun to receive scientific attention. Recently, the technical advances in headspace volatile collection has allowed standard scientific research on floral scent, resulting in many exciting discoveries [3, 4]. Simultaneously, the sheer number of such scent molecules and their complexity confound us [5].

Terpenoids, the largest class of floral volatiles, mediate the communication between plants and pollinators, herbivores, and pathogens [6–8]. All terpenoids are derived from two basic modules, isopentenyl diphosphate and its allyl isomer dimethylpropenyl diphosphate, which can be synthesized in plants via two compartmentalized pathways, namely the cytosolic mevalonic acid and plastidial methylerythritol phosphate pathways. Then, the two C5-isoprenes above are catalyzed by short-chain isoprenyl transferases to synthesize the substrate of a large class of terpene synthases (TPSs)/cyclases, namely geranyl diphosphate (GPP), farnesyl diphosphate (FPP), and geranyl geranyl diphosphate (GGPP). Thereafter, these building blocks are involved in isoprenoid biosynthesis within different subcellular compartments in plants.

The TPSs, a well-described family, catalyze the formation of the tremendous diversity of volatile terpenoids in plants. The TPSs are generally divided into seven clades, in which clades TPS-a, TPS-b, and TPS-g are angiosperm-specific clades responsible for the production of sesquiterpenes, cyclic monoterpenes, and acyclic mono- and sesquiterpenes, respectively [9]. To date, an increasing number of TPSs have been identified from plants, with about one-third isolated from ornamental plants. Specifically, flower-specific TPSs from *Clarkia*, snapdragon, rose, and *Osmanthus* have been isolated and identified. They catalyzed the formation of the monoterpene linalool, *β*-ocimene, *α*-pinene, myrcene, d-limonene, geraniol, and 1,8-cineole, as well as the sesquiterpenes nerolidol, *α*-farnesene, germacrene D, and aromadendrene [10]. Interestingly, some alternative metabolic pathways have been discovered in recent years. An unexpected enzyme—a Nudix hydrolase, RhNUDX1—shows geranyl diphosphate diphosphohydrolase activity, which is responsible for the formation of geraniol in roses [3]. Additionally, a new group of microbial-type *TPS*-like genes have been identified from non-seed plants [11].

**Figure 1:**
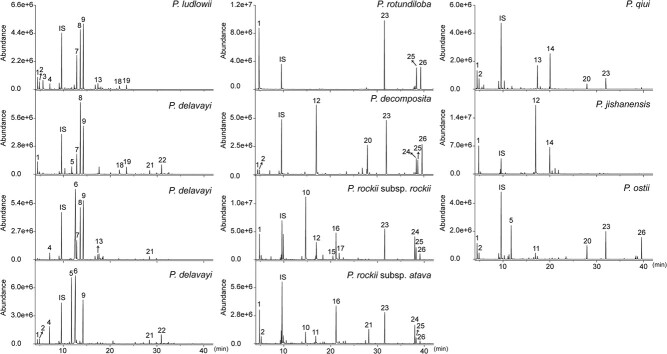
Chemical profiles of 11 wild tree peony genotypes. The volatiles of 11 wild tree peony genotypes collected from petals of initially opened flowers were profiled by GC–MS. All peaks were identified by reference to the NIST 14 library, in which peaks 2–12 and 15 were confirmed by comparison with the corresponding authentic standards. The dominant compounds identified from subsect. *Delavayanae* were (*Z*)-linalool oxide (peak 7), (*E*)-linalool oxide (peak 8), and linalool (peak 9), but these were absent in subsect. *Vaginatae*. IS, internal standard. Compound identification: 1, 2-hexenal; 2, 3-hexenol; 3, 1-hexanol; 4, 1*R*-α-pinene; 5, (*E*)-β-ocimene; 6, acetophenone; 7, (*Z*)-linalool oxide; 8, (*E*)-linalool oxide; 9, linalool; 10, 2-phenylethanol; 11, (*E*)-2-nonenal; 12, 1,4-dimethoxybenzene; 13, ethyl benzoate; 14, β-citronellol; 15, neral; 16, geraniol; 17, geranial; 18, (*Z*)-cinnamaldehyde; 19, (*E*)-cinnamaldehyde; 20, 1,3,5-trimethoxybenzene; 21, β-caryophyllene; 22, germacrene D; 23, pentadecane; 24, 6,9-heptadecadiene; 25, 8-heptadecene; 26, heptadecane.

Tree peony, as a more primitive taxa of *Paeonia*, originated in the Pan-Himalaya, migrated north-eastwards to the Hengduan Mountains region and further to the Qinba Mountain area, and diverged into two branches, subsect. *Delavayanae* and subsect. *Vaginatae*. There are nine wild species from section *Moutan*, and two species from subsect. *Delavayanae* distributed in the Pan-Himalayan region, while seven species from subsect. *Vaginatae* are mainly located in the Qinba Mountains. It is recorded that cultivated tree peonies (*Paeonia × suffruticosa*) originated in China more than 1400 years ago and were further introduced to Japan, Europe, and North America, resulting in two major cultivated groups, *P. × suffruticosa* from China and *P. × suffruticosa* from Japan, respectively [12]. Thereinto, independent domestications occurred in tree peonies with reddish-purple blotches at the base of the petals, widely cultivated in the middle Gansu Province, hereinafter referred to *Paeonia rockii* cultivars [13]. It has been documented that three cultivated groups originated from interspecific hybridization between five wild species from subsect. *Vaginatae* [14]. *Paeonia delavayi* and *Paeonia lutea*, later merged into one species, *P. delavayi*, were discovered in China in the late 19th century, and then introduced to Europe and North America [15]. French breeders, Lemoine and Henry, successfully created an entirely different group, called lutea hybrids, by crossing *P. delavayi* with *P. × suffruticosa* from China [16]. However, most *F*_1_ hybrids lacked stem hardiness with the lutea hook. Of significance, Saunders utilized *P. × suffruticosa* from Japan rather than *P. × suffruticosa* from China to produce large numbers of seedlings, which possess superior traits for flower color and stem hardiness. With the efforts of subsequent breeders, advanced-generation lutea hybrids with high fertility, hardier stems, and disease resistance were produced [16]. *P. delavayi* hybrids are now the most popular tree peonies in the world; they are held in high regard for their outstanding qualities and are widely planted in gardens and parks in East Asia, Europe, North America, New Zealand, and Melbourne, Australia.

In recent years, researchers have investigated the floral volatiles of wild tree peony species and some cultivars, whereas the flavor-related volatiles and the underlying molecular mechanism have not been adequately addressed [17, 18]. Additionally, transcriptome sequencing of three wild species and five cultivars has been performed to reveal the biosynthesis pathway genes for floral volatiles of tree peony, but none of the referred genes have been identified [19, 20]. We previously found that linalool may confer fragrance traits on *P. delavayi* hybrids [17]. However, total characterization of volatile blends of tree peony and the divergence of floral scent necessitates more typical cultivars, and the underlying mechanism remains unclear. Herein, we firstly profiled the volatile blends of tree peony and validated that linalool is a specific compound in subsect. *Delavayanae* and its hybrids. Furthermore, two TPSs (PdTPS1 and PdTPS4) that catalyze the formation of linalool were identified and characterized.

**Figure 2:**
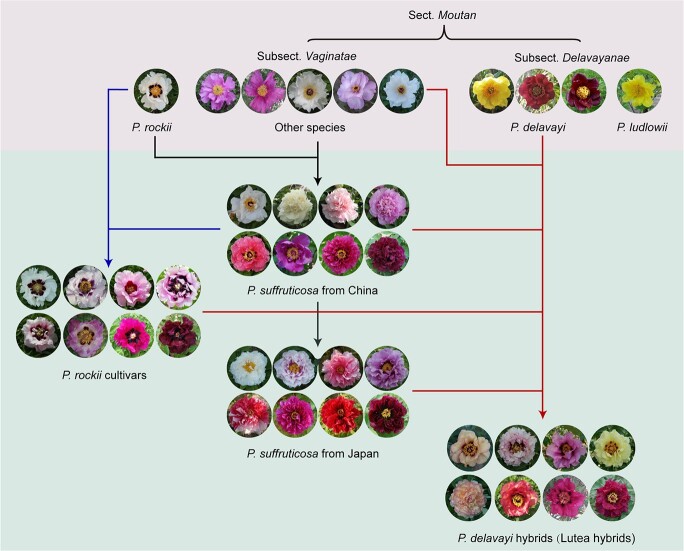
Relationship between wild species and cultivars of sect. *Moutan*. The wild species and cultivars are highlighted with different backgrounds.

## Results

### Linalool is a specific monoterpene in subsect. *Delavayanae*, but not in subsect. *Vaginatae*

To deeply understand the floral scent of tree peony, the volatiles from 11 wild tree peony genotypes were profiled by GC–MS (Fig. 1, Supplementary Data Table S1). A total of 56 volatiles were detected, in which the MS patterns of 22 compounds were fully consistent with their authentic standards. The detected compounds included 26 terpenoids (15 monoterpenoids, and 11 sesquiterpenoids), 20 fatty acid derivatives, nine benzenoids/phenylpropanoids, and one amino acid derivative (Supplementary Data Table S2). Noticeably, while significant differences in the volatile profiles were noted between subsect. *Delavayanae* and subsect. *Vaginatae*, the absolute level of some specific constituents between species varied by up to 50% or more (Fig. 1). For instance, linalool, a monoterpene, was evident in subsect. *Delavayanae*, yet absent in subsect. *Vaginatae*. As we all know, the floral scent of subsect. *Delavayanae* is quite different from that of subsect. *Vaginatae*. Linalool has been determined as a main contributor to the floral scent and fruit flavor of *Freesia* × *hybrida*, sweet osmanthus, wintersweet, and peach, as well as tree peony flowers [20–24]. In the course of development of tree peony flowers, the amount of linalool increased gradually and peaked at the fully opened stage [25]. Therefore, we supposed that linalool was a characteristic aroma component and played a crucial role contributing to the floral scent in subsect. *Delavayanae* as well as *P. delavayi* hybrids.

More specifically, terpenoids were the dominant volatiles in four samples from subsect. *Delavayanae*, and constituted 82.2% and 88.7% of the total volatiles in *P. ludlowii* (W04) and *P. delavayi* with purple-red flowers and anthers (W02), respectively (Fig. 1). The crucial compounds were linalool, (*E*)-linalool oxide, and (*Z*)-linalool oxide. Benzenoids/phenylpropanoids were also abundant in *P. delavayi* with yellow flowers (W01) and *P. delavayi* with purple-red flowers and yellow anthers (W03) due to a substantial proportion of detected acetophenone. Additionally, a high content of β-ocimene was detected in *P. delavayi* with purple-red flowers and yellow anthers. By contrast, alkanes, such as pentadecane, heptadecane, and 6,9-heptadecadiene, tended to accumulate in subsect. *Vaginata* except for *P. jishanensis* (W10). As a result, six species in subsect. *Vaginata* tended to accumulate abundant fatty acid derivatives, which were predominant in *P. rotundiloba* (W05), comprising about 98.2% of the total volatiles, followed by *P. rockii* subsp. *atava* (70.0%, W08) and *P. ostii* (70.1%, W11). Abundant 1,4-dimethoxybenzene and 1,3,5-trimethoxybenzene resulted in the high proportion of benzenoids/phenylpropanoids in *P. decomposita* (W06). It is well known that traditional landraces and cultivars from *P. rockii* emit a pleasant floral scent, but the aroma compounds are still unknown. Higher levels of 2-phenylethanol and geraniol were observed in *P. rockii* subsp. *rockii* and *P. rockii* subsp. *atava*, which were reported as important scent compounds in numerous flowers such as rose and petunia [3, 4]. The results implied that 2-phenylethanol and geraniol might be the flavor-related volatiles in the specific floral scent [20]. The total volatile content was the lowest in *P. qiui* (W09), in which β-citronellol and ethyl benzoate were the characteristic components. Strikingly, 1,4-dimethoxybenzene was highest in *P. jishanensis* and, to a lesser extent, citronellol. Notably, β-ocimene, a monoterpene, was evident in *P. ostii*.

### Tree peony cultivars emit heterogeneous floral terpenes, while linalool is widespread in *P. delavayi* hybrids

Generally, cultivated tree peonies include four groups: (1) traditional cultivars, *P. × suffruticosa*, from China, originating from homoploid hybridization between five wild woody species [14]; (2) *P. × suffruticosa* from Japan, domesticated from Chinese *P. × suffruticosa*; (3) *P. rockii* cultivars with reddish-purple blotches at the base of the petals; and (4) *P. delavayi* hybrids (lutea hybrids), a new group from a distant hybridization between subsect. *Delavayanae* and subsect. *Vaginatae* (Fig. 2). Linalool is a characteristic aroma component in subsect. *Delavayanae*, and whether it is retained in *P. delavayi* hybrids is an unresolved question deserving attention. We systemically investigated the volatile profiles from various cultivated tree peonies, comprising 22 *P. × suffruticosa* from China, 15 *P. × suffruticosa* from Japan, 17 *P. rockii* cultivars and 43 *P. delavayi* hybrids (Supplementary Data Table S1). The results showed that tree peony cultivars emitted heterogeneous volatiles, in which terpenoids were the dominant volatiles of *P. delavayi* hybrids, ranging from 34.4% to 96.2% of the total volatiles. Conversely, *P. × suffruticosa* and *P. rockii* cultivars tended to accumulate abundant fatty acid derivatives, and the main compounds were analogous to those of the wild species.

**Figure 3:**
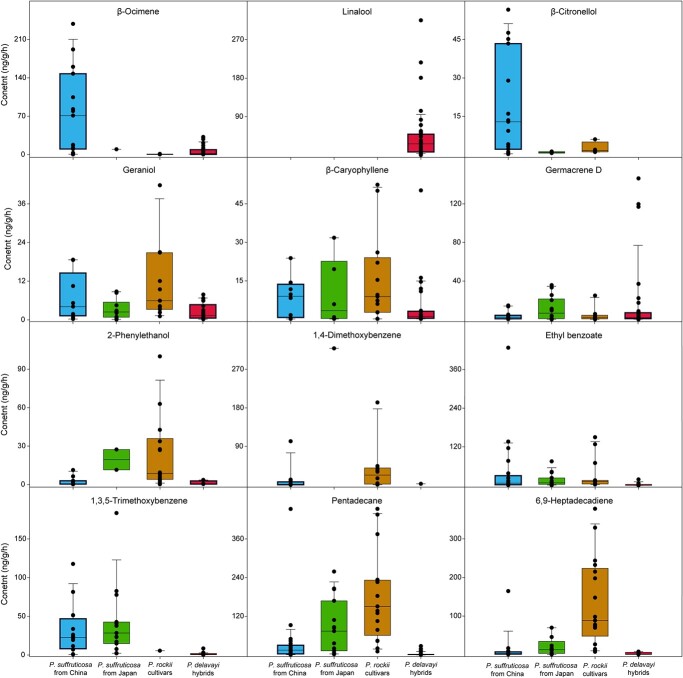
The range and distribution of representative volatiles released from tree peony cultivars. Cultivars were divided into four groups depending on genetic background, comprising 22 *P.* × *suffruticosa* from China, 15 *P.* × *suffruticosa* from Japan, 17 *P. rockii* cultivars, and 43 *P. delavayi* hybrids.

Specifically, 85 quantifiable volatiles were detected in tree peony cultivars (Supplementary Data Table S2). As shown in Fig. 3 and Supplementary Data Fig. S1, the leading volatile terpenes between cultivars differed; there were higher contents of β-ocimene, linalool, β-citronellol, geraniol, β-caryophyllene, and germacrene D , followed by α-pinene, neral, and geranial. Noticeably, linalool was widespread in *P. delavayi* hybrids, but absent in other groups. Although linalool was the dominant terpene compound in the majority of *P. delavayi* hybrids, the contents varied dramatically, accounting for an estimated 1.1%–80.5% of the total volatiles. And linalool accounted for <20.0% of total volatiles in only nine *P*. *delavayi* hybrids. Additionally, we found that 2-phenylethnol was a characteristic compound in *P. rockii* cultivars, and 1,3,5-trimethoxybenzene was typical of *P. × suffruticosa*, while ethyl benzoate, pentadecane, and 6,9-heptadecadiene was of higher content in almost all cultivars except for *P. delavayi* hybrids.

### Terpene synthases mediate the differential volatile emissions between cultivars

To explore the underlying mechanism of tree peony floral scent, we combined next-generation sequencing (NGS) with single-molecule long-read isoform sequencing (SMRT-seq). ‘High Noon’ is a global representative *P. delavayi* hybrid with a typical floral scent, which was selected to elucidate linalool biosynthesis in tree peony. Firstly, the full-length isoform sequencing (Iso-Seq) of ‘High Noon’ was performed with PacBio Sequel II. A total of 25.72 Gb clean data were generated, and we finally obtained 58 153 non-redundant transcripts after the modification of site mismatch, in which 47 686 transcripts were annotated by aligning to public databases. To more thoroughly investigate the putative TPSs, the annotated tree peony proteome was searched with 45 TPS protein sequences of *Arabidopsis* and two Pfam models (PF01397 and PF03936), which referred to the C and N terminal domain of TPSs, respectively. As a result, a total of 48 putative TPSs were identified in combination with hidden Markov model (HMM) and BLASTP programs. The above 48 sequences were manually screened to remove identical sequences or incorrectly assembled sequences. Of these, 26 were likely to encode full-length TPS proteins.

**Figure 4:**
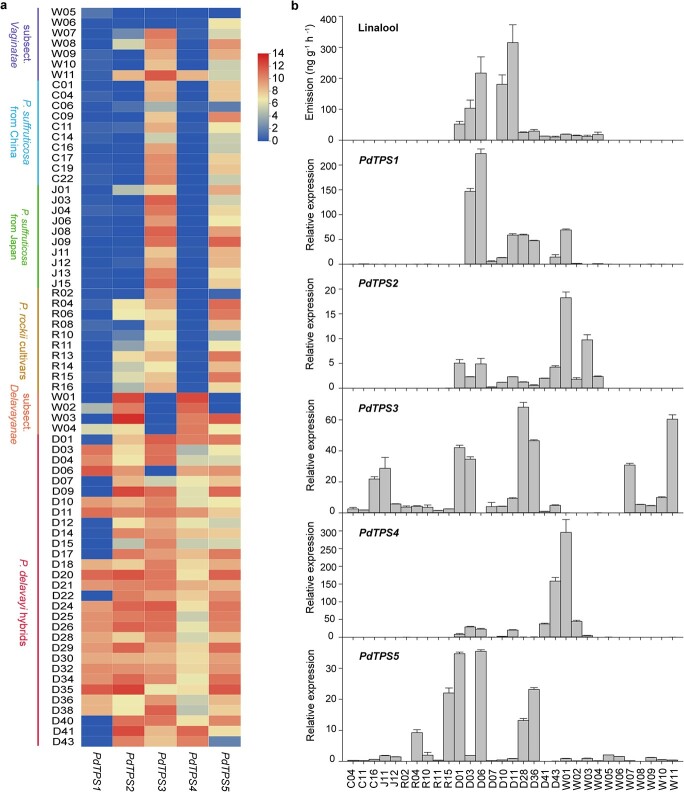
Correlation between transcript levels of *PdTPS1* and *PdTPS4* and content of linalool in tree peony. **a** Expression patterns of five candidate genes encoding TPSs is represented by color gradations. The expression data were obtained from RNA-seq analysis, involving 4 species from subsect. *Delavayanae*, 7 species from subsect. *Vaginatae*, 10 *P.* × *suffruticosa* from China, 10 *P.* × *suffruticosa* from Japan, 10 *P. rockii* cultivars, and 30 *P. delavayi* hybrids. **b** Contents of linalool and relative expression levels of *PdTPS1* and *PdTPS4* across tree peony cultivars and species were determined using RT–qPCR and calculated by 2^−ΔCτ^ in triplicate.

Then, we sequenced the petal transcriptomes of fully opened tree peony flowers with exposed anthers to collect the gene expression profile, involving 11 wild genotypes, 30 *P. delavayi* hybrids, 10 *P. × suffruticosa* cultivars from China, 10 *P. × suffruticosa* cultivars from Japan, and 10 *P. rockii* cultivars. The gene expression levels of 26 TPSs were obtained by mapping all RNA-seq reads to the full-length transcriptome. As a result, F01_transcript_52927, F01_transcript_10576, and F01_transcript_9634 were highly expressed in a substantial proportion of *P. delavayi* hybrids and four wild genotypes in subsect. *Delavayanae*, while F01_transcript_98869 and F01_transcript_42388 exhibited high expression in almost all samples (Fig. 4a, Supplementary Data Fig. S2). Therefore, the above five transcripts were candidates that resulted in the biosynthesis of the specific monoterpene linalool, and we designated these gene models as *PdTPS1* through *PdTPS5*.

**Figure 5:**
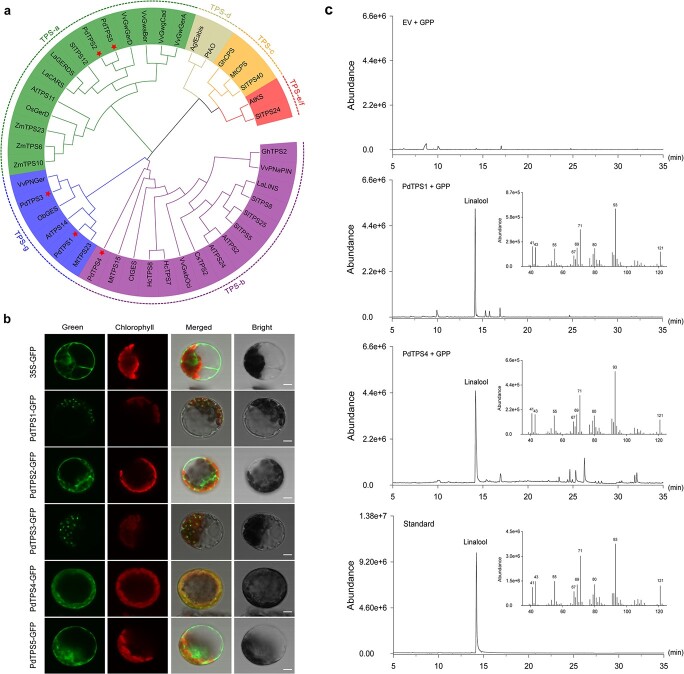
Characterization of TPS proteins from tree peony. **a** Phylogenetic analysis of TPS proteins from tree peony and other plants. The phylogenetic tree was constructed with 1000 bootstrap replicates using the maximum likelihood method based on the Jones-Taylor-Thornton (JTT) matrix-based model using MEGAX software. Five tree peony TPS proteins in this study are highlighted by stars. The TPS-a, TPS-b, TPS-c, TPS-d, TPS-e/f, and TPS-g clades are highlighted with colored lines and backgrounds. Detailed information on TPS proteins is provided in Supplementary Data Table S4. **b** Subcellular localization of the five PdTPS–GFP fusions in *Arabidopsis* leaf protoplasts. Green, GFP fluorescence of each fusion protein; Chlorophyll, chlorophyll autofluorescence; Merged, merged green and red channel images; Bright, bright-field image. Scale bars, 20 μm. **c** Functional characterization of the monoterpene synthases PdTPS1 and PdTPS4. Products of the heterologously expressed enzymes of empty vector, PdTPS1, and PdTPS4 using GPP as substrate were determined by GC–MS. The GC–MS spectrum of linalool standard was used as reference.

### *PdTPS* genes showed different expression patterns between tree peony cultivars

Transcriptome sequencing revealed that five genes encoding TPSs were significantly upregulated in *P. delavayi* hybrids. Then, we performed qRT–PCR to investigate the expression patterns of five candidates among 11 wild genotypes, 10 *P. delavayi* hybrids and 10 cultivars without *P. delavayi* lineage (*P. × suffruticosa* and *P. rockii* cultivars). In agreement with the patterns of linalool emission, *PdTPS1*, *PdTPS2*, and *PdTPS4* were highly expressed in *P. delavayi* hybrids and wild species from subsect. *Delavayanae* (Fig. 4b), implying their involvement in the biosynthesis of linalool. Interestingly, it was observed that *PdTPS1* and *PdTPS4* showed completely different expression patterns. *PdTPS1* transcripts were detected at higher levels in *P. delavayi* hybrids, whereas *PdTPS4* exhibited relatively higher expression levels in wild species of subsect. *Delavayanae* (Fig. 4b). In contrast, the expression of *PdTPS3* and *PdTPS5* did not show this pattern, which suggested that they may participate in the formation of other terpenes rather than linalool (Fig. 4b). However, much-needed evidence is imperative to elucidate the catalytic properties of the PdTPS proteins.

### The candidate terpene synthases are ascribed to three angiosperm-specific terpene synthase clades

Firstly, the five putative genes encoding TPSs mentioned above were amplified from ‘High Noon’. The putative proteins ranged from 553 to 600 amino acid residues and had a calculated molecular mass of 65 kDa (Supplementary Data Table S3). The deduced amino acid sequences of these genes contained C-terminal DDXXD and NSE/DTE motifs. PdTPS2, PdTPS4, and PdTPS5 had an obvious twin-arginine RR(X)_8_W motif responsible for monoterpene cyclization at the N-terminal end (Supplementary Data Fig. S3), which is also conserved in most sesquiterpene and diterpene synthases [26]. Full-length cDNA sequences of five putative genes were simultaneously obtained from seven tree peony cultivars, comprising five *P. delavayi* hybrids (emitting linalool) and two *P. × suffruticosa* (not emitting linalool). One gene from different cultivars showed high amino acid sequence identities ranging from 98.4% to 100%. Remarkably, the amino acid sequences of PdTPS1 and PdTPS4 from two *P. × suffruticosa* (‘Bai He Zhan Chi’ and ‘Tian Xiang’) and two *P. delavayi* hybrids (‘High Noon’ and ‘Okan’) were identical (Supplementary Data Figs S4–S8).

Phylogenetic analysis was conducted by aligning the amino acid sequences of the five putative functional PdTPS proteins together with other previously identified plant TPSs (Fig. 5a, Supplementary Data Table S4). The result demonstrated that five PdTPSs identified in this study were ascribed to three angiosperm-specific clades: TPS-a, TPS-b, and TPS-g. Specifically, PdTPS2 and PdTPS5 were clustered into the TPS-a clade, most of which are sesquiterpene synthases. PdTPS4 was grouped into the TPS-b clade, which is characterized as either monoterpene synthases or isoprene synthases and is specific to flowering plants. PdTPS1 and PdTPS3 were grouped into the TPS-g clade, which is more prone to producing acyclic monoterpenes [9]. Subcellular localization analysis indicated that PdTPS1, PdTPS3, and PdTPS4 were localized in the plastids, whereas PdTPS2 and PdTPS5 were in the cytoplasm (Fig. 5b). Thus, we predicted that PdTPS1 and PdTPS4 were among the best candidates to catalyze linalool biosynthesis taking into consideration the gene expression patterns.

### PdTPS1 and PdTPS4 recombinant proteins reveal the catalytic activity of linalool synthase

To initially characterize the synthases encoded by the *PdTPS* genes, their biochemical properties were investigated. Full-length cDNAs for five *PdTPS* candidates (*PdTPS1* to *PdTPS5*) were isolated and heterologously expressed in *Escherichia coli* for the production of recombinant proteins. The polyhistidine-tagged crude lysates were then purified with a nickel-affinity column and confirmed by western blot analysis (Supplementary Data Fig. S9). The activities of purified recombinant PdTPS proteins were analyzed by using a number of potential substrates including GPP, neryl pyrophosphate (NPP), *E*,*E*-FPP, *Z*,*Z*-FPP, and GGPP. The products of the enzyme assays were analyzed by GC–MS and identified by comparing mass spectra with corresponding standards.

Generally, three monoterpene synthases from tree peony were found to be single-product enzymes, whereas two sesquiterpene synthases exerted their versatile and diverse functions (Supplementary Data Table S5). The PdTPS1, PdTPS3, and PdTPS4 recombinant proteins catalyzed the biosynthesis of monoterpenes *in vitro* with GPP as substrate. Consistent with a previous hypothesis, both PdTPS1 and PdTPS4 catalyzed the formation of acyclic terpene alcohols with linalool as the single product (Fig. 5c), which is the dominant flavor-related volatile in subsect. *Delavayanae* and *P. delavayi* hybrids. The reaction products catalyzed by PdTPS3 with GPP formed geraniol rather than linalool, which exhibited high expression levels in almost all tree peony cultivars. The other two characterized *PdTPS* genes encoded multi-product sesquiterpene synthases, and generated nearly all of the sesquiterpenes detected in tree peony, which is typical of many plant sesquiterpene synthases [27]. Comparison of enzyme activities at different substrate concentrations showed that there was no significant difference in enzyme activity between PdTPS1 and PdTPS4 (Supplementary Data Table S6).

### Functional characterization of *PdTPS1* and *PdTPS4* genes *in planta* was consistent with products obtained *in vitro*

**Figure 6:**
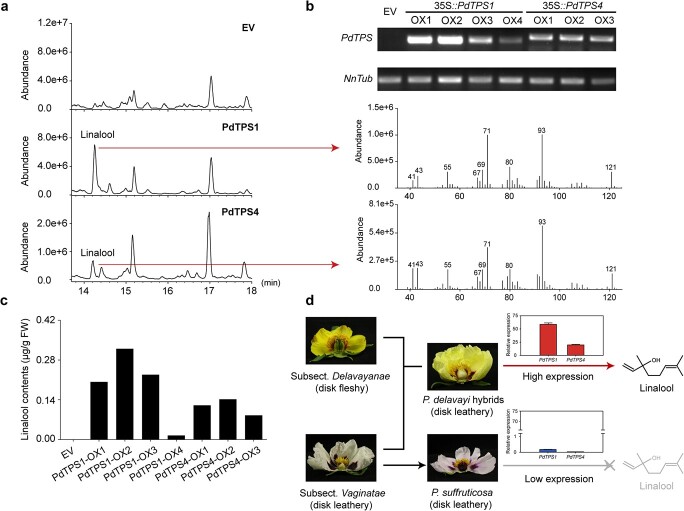
Functional characterization of PdTPS1 and PdTPS4 *in vivo*. **a** Volatiles detected in transgenic tobacco lines and empty vector control. **b** Expression analysis of *PdTPS1*and *PdTPS4* by RT–PCR in empty vector and *PdTPS1* and *PdTPS4* transgenic lines, respectively. **c** Contents of linalool detected in PdTPS1 and PdTPS4 transgenic lines. **d** A proposed model for linalool biosynthesis in flowers of tree peony. Briefly, the flavor-related volatiles of sect. *Moutan* are highly divergent, and linalool is a specific monoterpene in subsect. *Delavayanae*. PdTPS1 and PdTPS4 catalyze the formation of linalool, which are abundantly expressed in subsect. *Delavayanae* but not in subsect. *Vaginatae* with typical *P. delavayi* hybrid ‘High Noon’ and *P. × suffruticosa* ‘Bai He Zhan Chi’ as materials. Their species-specific and cultivar-specific expression patterns may result in the differential accumulation of linalool in tree peony.

To further investigate the enzymatic function of PdTPS1 and PdTPS4 *in planta*, we used *Agrobacterium*-mediated transformation of tobacco for *in vivo* studies. The volatile profiles of transgenic and control lines were analyzed by GC–MS. The major products detected in the transgenic tobacco *in planta* were consistent with recombinant proteins *in vitro*. Specifically, four 35S::*PdTPS1* and three 35S::*PdTPS4* lines all accumulated linalool. In contrast, no linalool was detected in the control lines (Fig. 6a). The four 35S::*PdTPS1* and three 35S::*PdTPS4* transgenic lines and the three control lines were found to accumulate similar mRNA levels of *PdTPS1* and *PdTPS4* (Fig. 6b), respectively. These results further confirmed the enzymatic activity of PdTPS1 and PdTPS4, and the differential expression resulted in the species-specific and cultivar-specific emission of linalool in tree peony (Fig. 6c).

## Discussion

### Linalool may have experienced selective pressure to facilitate the adaptability of *Paeonia*

Tree peonies are stunning horticultural plants widely cultivated in temperate regions, especially in East Asia, Europe, and North America. All species are divided into two subsections, *Delavayanae* and *Vaginatae*, which have distinct floral scent and morphological characters as well as geographical distribution. Specifically, subsect. *Delavayanae* consists of two species, *P. ludlowii* and *P. delavayi*, which are restricted to the south-eastern Tibetan Plateau and the north-west Yunnan-Kweichow Plateau at a higher altitude range. Conversely, wild species from subsect. *Vaginatae* have a wider geographical distribution in the Hengduan Mountains and Qinba Mountains [28]. Floral scent is important for understanding of the adaptations and evolution of flowering plants [29]. Azalea is one striking example of floral scent evolution. *Rhododendron* is a typical alpine plant and contains two distinct groups. The genome of *R. ovatum*, the representative species distributed in low altitudes with broad adaptation, contains many more TPS genes in comparison with two high-altitude *Rhododendron* species, which play a major role in floral scent biosynthesis and defense responses [30]. In this study, the floral-scent bouquets of wild tree peony species were investigated. The results suggested that the predominant volatiles were significantly more enriched in subsect. *Vaginatae* than in subsect. *Delavayanae* (Supplementary Data Table S2). Therefore, we speculate that the enriched volatiles in subsect. *Vaginatae* may account for its high levels of environmental adaptability. More importantly, scent is shaped by differential selection pressures in *Penstemon digitalis*, which benefits reproductive success in turn. Linalool has been proved to be a direct target of selection [31]. Similarly, linalool is widespread in subsect. *Delavayanae* rather than subsect. *Vaginatae*, suggesting its experience of selective pressure in tree peony.

Floral scent constitutes an ancient and important channel of communication between flowering plants and their pollinators or antagonist [32]. Tree peony is a typical entomophilous plant with a wide variety of pollinating insects, but studies identifying pollinators to species level are rare. To date, a few studies indicated that bees were the most common pollinators and there is no difference in pollinators between subsect. *Delavayanae* and subsect. *Vaginatae* [33]. In this study, we found that the floral volatiles of the two subsections were entirely different, and further studies on the pollination biology of *Paeonia* would further our understanding of plant–pollinator relationships. On the other hand, some volatiles are poisonous to herbivores. For example, linalool exhibited strong repellent ability towards non-pollinating ants to prevent their consuming nectar and caused oviposition avoidance behavior of lepidopteran insects to reduce the number of enemies [8, 34]. Interestingly, the wild species of subsect. *Delavayanae* have nectaries at the base of the disk, which can secrete nectar. Conversely, nectaries are absent in subsect. *Vaginatae*, and pollen is the only pollination reward. Therefore, we speculated that linalool may play a major role in regulating tree peony defense responses against various herbivores and pathogens. Further studies of pollinator- and antagonist-mediated selection on this compound will facilitate our understanding of scent evolution in *Paeonia*.

### Terpene synthases mediate a tremendous diversity of volatile terpenes in tree peony

Floral scent results from a tremendous diversity of volatiles, which confer the flowers’ unique fragrance. Generally, the volatile profiles vary remarkably among species and even cultivars within the same species. It has been proved that the diversity of volatile terpenes in plants may be ascribed to a mid-size family of TPSs, which have clearly diverged in different lineages [9]. However, the floral scent compounds of tree peony have yet to be adequately addressed. In this study, volatiles from 11 wild tree peony genotypes and 97 cultivars were specially profiled. A wide range of floral terpenes were detected, comprising 18 monoterpenes, 19 sesquiterpenes, and 2 irregular terpenes (Fig. 1, Supplementary Data Fig. S1). The main floral scent volatiles in tree peony are terpenoids and vary dramatically between species and cultivars, including linalool, β-ocimene, β-citronellol, geraniol, β-caryophyllene, and germacrene D (Fig. 3). Strikingly, all species of subsect. *Delavayanae* have a sweet floral scent, in which *P. delavayi* is widely used as a breeding parent for its scent phenotype and its offspring are called *P. delavayi* hybrids (lutea hybrids). Analogously, linalool has been found to be synthesized only in subsect. *Delavayanae* rather than subsect. *Vaginatae* [20]. Generally, species of subsect. *Delavayanae* and their hybrids exhibit floral scent phenotypes distinct from those of *P. × suffruticosa* and *P. rockii* cultivars. It has been proved that linalool is the dominant volatile monoterpene in the floral scents of many ornamental plants, such as *Clarkia breweri*, wintersweet (*Chimonanthus praecox*), and *Freesia* × *hybrida* [21, 23, 35]. Thus, we suppose that linalool may play a crucial role in the typical floral scent of *P. delavayi* hybrids. In contrast, the floral sent compounds in the species of subsect. *Vaginatae* and their offspring were heterogeneous.

Generally, monoterpenes are synthesized by the plastidial pathway, while sesquiterpenes are synthesized via the cytosolic pathway. The five TPSs from tree peony provide no exception to the rule. Three monoterpene synthases (PdTPS1, PdTPS3, and PdTPS4) were shown to be localized in plastids, while two sesquiterpene synthases (PdTPS2, and PdTPS5) were localized in the cytosol (Fig. 5b). The species-specific and cultivar-specific expression patterns of *PdTPS1* and *PdTPS4* are supposed to be the determinant factors causing the cultivar-specific emission profiles. Interestingly, we observed that linalool was present in all species from subsect. *Delavayanae* and almost all the *P. delavayi* hybrids. Thus, we supposed that linalool biosynthesis might be inherited. Obviously, we noted a generally higher content of linalool in advanced-generation lutea hybrids. Further research was needed to elucidate the underlying mechanism. Nonetheless, wild species from subsect. *Delavayanae* contains rich genetic diversities and excellent genes encoding terpenes for improvement of floral scent.

### Transcription of terpene synthases is strictly controlled at the transcript and protein levels

As shown in Figs 1 and 3, linalool was the predominant compound in four wild genotypes from subsect. *Delavayanae* and its hybrids, but absent in subsect. *Vaginatae*, *P. × suffruticosa*, and *P. rockii* cultivars. Interrogation of the comprehensive collection of transcriptome data from tree peony revealed that *PdTPS1* and *PdTPS4* are expressed more highly in species from subsect. *Delavayanae* and its hybrids, which is consistent with the emission patterns of linalool (Figs 3 and 4a). In contrast, *PdTPS1* and *PdTPS4* are not expressed or poorly expressed in other tree peony groups. But how the precise regulation of TPSs responsible for linalool biosynthesis is achieved is yet to be clarified. The multiple sequence alignment results indicated that almost all TPSs showed high identities between cultivars. For instance, *PdTPS1* and *PdTPS4* genes were amplified from total RNA isolated from several tree peony cultivars. Surprisingly, the amino acid sequences of *PdTPS1* and *PdTPS4* from two *P. delavayi* hybrids (emitting linalool) and two *P. × suffruticosa* (not emitting linalool) are identical (Supplementary Data Figs S4 and S7). Thus, we speculated that the divergent accumulation of linalool in tree peony was not caused by differences in amino acid sequence. The transcription of *PdTPS1* and *PdTPS4* genes might be strictly controlled by a transcriptional mechanism.

Linalool is widely detected across the plant kingdom. Researchers have made great progress in identifying linalool synthase genes in recent decades, but much less is known about the regulation mechanism [36–39]. So far, there are only a few demonstrated regulatory networks. It was reported that an MYB–bHLH complex (FhMYB21 and FhMYC2) mediate the expression level of the linalool synthase gene *FhTPS1* in flowers of *Freesia hybrida* [40]. The methyl jasmonate-responsive transcription factor DobHLH4 has been proved to be a positive regulator of linalool biosynthesis in *Dendrobium officinale* [41]. Recently, PpbHLH1 was demonstrated to directly activate the expression of *PpTPS3* in peach fruit [24]. The expressions of linalool synthases PpTPS1 and PpTPS3 were activated by transcription factor PpERF61 [42]. Thus, what emerges is that the regulation of linalool synthase genes is a complex transcriptional network, and the fluxes can be finely coordinated at multiple levels. Strikingly, we observed that *PdTPS4* exhibited a higher expression than *PdTPS1* in wild species of subsect. *Delavayanae*, while it was actually quite the opposite in *P. delavayi* hybrids (Fig. 4b). We speculated that the expression of *PdTPS1* was inhibited in wild species of subsect. *Delavayanae*, which was partially relieved after hybridization. However, much less is known about regulation of linalool biosynthesis, which remains to be investigated in more detail.

In conclusion, the blends of volatiles in tree peony were characterized, and the biogenesis of flavor-related linalool distinguished subsect. *Delavayanae* and its hybrids from subsect. *Vaginatae*. Further biochemical evidence found that PdTPS1 and PdTPS4 catalyzed the production of flavor-related linalool. The differential expression of *PdTPS1* and *PdTPS4* is likely to result in the divergent biosynthesis of linalool between subsect. *Delavayanae* and subsect. *Vaginatae*. This study revealed the underlying mechanism of the floral scent divergence in tree peony and provides a critical basis for understanding the speciation and fitness of *Paeonia*.

## Materials and methods

### Plant materials and chemical reagents

The total of 11 accessions from eight wild tree peony species and 97 cultivars derived from various genetic backgrounds were sampled in this study (Supplementary Data Table S1). Ten wild tree peony genotypes and three *P. delavayi* hybrids, including *P. delavayi* with yellow flowers (W01), *P. delavayi* with purple-red flowers and anthers (W02), *P. delavayi* with purple-red flowers and yellow anthers (W03), *P. ludlowii* (W04), *P. decomposita* (W05), *P. rockii* subsp. *rockii* (W07), *P. rockii* subsp. *atava* (W08), *P. qiui* (W09), *P. jishanensis* (W10), *P. ostii* (W11), ‘Huang Shui Jing’, ‘Zi Yan Gui Chao’, and ‘Mei Xiang Hong’, were grown at Gansu Forestry Science and Technology Extension Station (Lanzhou, Gansu, China). *P. rotundiloba* (W06) was collected from Lixian County, Sichuan, China. The other 27 *P. delavayi* hybrids were collected from Jiu Feng Forestry Experiment Station of the Beijing Forestry University in China, and five *P. delavayi* hybrids were obtained from the National Chinese Botanical Garden, Beijing. The remaining tree peony cultivars were cultivated at the Institute of Botany, Chinese Academy of Sciences. Three replicates were prepared for each sample. *Arabidopsis thaliana* and *Nicotiana tabacum* plants were grown under light (dark) cycles of 16 (8) hours at 22°C.

(−)-α-Pinene, β-ocimene, (*Z*)-linalool oxide, (*E*)-linalool oxide, (−)-linalool, 2-phenylethanol, ethyl benzoate, 1,4-dimethoxybenzene, β-citronellol, neral, geraniol, geranial, 1,3,5-trimethoxybenzene, β-caryophyllene, pentadecane, C7–C40 saturated alkanes standard mix, GPP, NPP, *E,E*-farnesyl pyrophosphate (*E,E*-FPP), GGPP, and 3-octanol were obtained from Sigma–Aldrich (Shanghai, China), while *Z,Z*-FPP was purchased from Echelon Biosciences (UT, USA). Germacrene D was obtained from Cayman Chemical Company (MI, USA). Chromatographic-grade hexane was purchased from Sigma–Aldrich (Shanghai, China).

### Volatile collections and GC–MS analysis of volatile compounds

Petals from fully opened flowers of wild species and cultivars were collected and immediately sealed into headspace bottles with a polytetrafluoroethylene septum, and 3-octanol was added as an internal standard. Headspace solid-phase microextraction was employed to collect the petal volatiles, which were extracted with a 50/30-μm DVB/CAR/PDMS fiber (Supelco, USA) for 0.5 hour at 40°C. Then, the enriched volatile compounds on the fiber were desorbed in the GC injector at 250°C for 3 minutes. The injection mode was split with a split ratio of 10:1. The analysis was carried out using an Agilent 7890B-7000C gas chromatography tandem triple quadrupole mass spectrometer. The capillary column was an HP-5MS column (5% phenyl methyl siloxane, 30 m × 0.25 mm i.d., 0.25 μm film thickness; Agilent). The temperature program started at 55°C for 3 minutes and then increased by 3°C per minute to 210°C. The MS conditions were as follows: transfer line temperature, 280°C; source temperature, 230°C; ionization potential, 70 eV; scan range, 30–600 amu. The volatile compounds were identified by matching the mass spectra with the NIST14 library as a reference, while the dominant compounds were confirmed by standards [17]. Tentative identifications of the other compounds were performed by comparing their retention indices (RIs) and MS spectra. RIs were calculated by analyzing the C7–C40 *n*-alkane under the same chromatographic conditions according to previously reported methods [43]. Relative quantification of each volatile was carried out using the peak area of the internal standard as a reference.

### Transcriptome sequencing

For Iso-Seq analysis, total RNA of flower, leaf, and stem tissues from different developmental stages of ‘High Noon’ were isolated with an RNAprep Pure Plant Kit (Tiangen, China), which were pooled in equal amounts for cDNA synthesis with the Clontech SMARTer™ PCR cDNA Synthesis Kit (Mountain View, CA, USA). Size fractionation and selection were carried out using the BluePippin Size Selection System (Sage Science, USA), in which fractions of 1–2, 2–3, and 3–6 kb were collected, and treated with DNA damage repair mix. The SMRT libraries were generated using the PacBio SMRTbell Template Prep Kit (Pacific Biosciences, USA) and sequenced by PacBio Sequel II.

For RNA-seq analysis, the petals of fully opened tree peony flowers with exposed anthers were collected, involving 11 wild tree peony genotypes, 30 *P. delavayi* hybrids, 10 *P. × suffruticosa* from China, 10 *P. × suffruticosa* from Japan, and 10 *P. rockii* cultivars. Total RNA was separately isolated with TRIzol Reagent (Invitrogen), and then quantified using a NanoDrop ND-2000 (NanoDrop Technologies). RNA-seq libraries were constructed separately using the VAHTS™ mRNA-seq V2 Library Prep Kit (Vazyme Biotech, China). Then, we performed paired-end (150PE) sequencing on an Illumina NovaSeq 6000. The raw data were firstly trimmed by Trimmomatic (v0.36) with the shortest read length of 90 bp. The cleaned reads were then mapped to the reference transcripts and the expression level was extracted by RSEM (v3).

### Identification of terpene synthases in tree peony

‘High Noon’, obtained by crossing *P. delavayi* with *P. × suffruticosa*, is one of the most popular intersubsectional hybrids cultivated worldwide, and has a sweet floral scent. The annotated proteome of ‘High Noon’ obtained by Iso-Seq analysis was searched with two Pfam models, PF01397 and PF03936, using the hmmsearch command in the HMMER package [44]. Additionally, 45 TPS protein sequences of *Arabidopsis* were used as queries in a BLASTP search against the annotated proteome of tree peony to obtain the candidates with high sequence similarity. Subsequently, we combined the two TPS gene sets above, and the domain of the candidates was then manually curated by the NCBI Conserved Domain Search.

### Cloning of full-length cDNA of candidate *PdTPS* genes and phylogenetic analysis

Total RNA was extracted from petals using the Plant RNA Kit (Omega) according to the manufacturer’s instructions. cDNA was synthesized using the FastKing gDNA dispelling RT SuperMix (Tiangen). The open reading frames (ORFs) were cloned with a 2 × Super Pfx MasterMix (CWBIO) by using the *PdTPS* forward/reverse primers (Supplementary Data Table S7) from *P. × suffruticosa* cv. ‘High Noon’. PCR products of appropriate length were cloned into the *pEASY*^®^-Blunt Cloning Vector (TransGen) and sequenced. Using the maximum likelihood method with 1000 bootstrap replicates, a phylogenetic tree was reconstructed with MEGAX with default parameters, and conserved regions were highlighted.

### Quantitative real-time PCR analysis

Flower petals from different wild genotypes and cultivars were sampled. Total RNA was prepared as described above. Quantitative assays of five candidate *PdTPS* genes were carried out by using HiPer Realtime PCR Super mix (Mei5Biot) and analyzed with ABI StepOnePlus™ (Applied Biosystems, USA) and performed in triplicate. The specific primer pairs of five candidate *PdTPS* genes are listed in Supplementary Data Table S7, and the gene *β-tubulin* was used as an internal control [45].

### Subcellular localization of PdTPS proteins

The coding sequences of the *PdTPS* genes without the stop codon were subcloned from *pEASY*^®^-Blunt Cloning Vector into the *pUC19* vector. The constructs were transfected into *Arabidopsis* protoplasts as described previously [46]. After incubation for 12–16 hours in darkness, the fluorescence was visualized using a multiphoton laser scanning microscope (Olympus FV 1000 MPE).

### Heterologous expression of candidate PdTPS proteins in *Escherichia coli* and *in vitro* enzyme assay

The sequenced cDNA of six candidate *PdTPS* genes was amplified and subcloned into the pEASY^®^-Blunt E1 Expression Vector (TransGen) to express proteins in *E. coli* strain *Transetta* (DE3). Liquid cultures of the bacteria harboring the empty vector and different *PdTPS* genes were grown at 37°C to an OD_600_ of 0.6. Recombinant proteins with a His(6) tag at the C-terminus were induced by the addition of 0.3 mM isopropyl β-d-1-thiogalactopyranoside (IPTG), and the cultures were incubated for 24 hours at 16°C. The cells were harvested by centrifugation at 10 000 × g for 4 minutes at 4°C, and disrupted by sonication on ice for 10 min in chilled assay buffer [50 mM HEPES, pH 7.5, 5 mM DTT, 10% (vol/vol) glycerol]. The supernatants were collected at 12 000 × g for 15 min at 4°C, and purified through an Ni-NTA agarose column (Qiagen, Germany). Finally, the purified proteins were concentrated and desalted into assay buffer by passage through an Amicon Ultra-15 Ultra 10 K (Merck Millipore). The purified proteins were confirmed by western blot via a WES Protein Simple system and quantified using Bradford Protein Assay Kit (Beyotime, Shanghai, China).

To determine the catalytic activity of PdTPSs, enzyme assays containing 50 μg purified proteins and 50 μl assay buffer with 10 μM GPP, FPP, or GGPP, 30 mM KCl, 5 mM MgCl_2_, and 1 mM MnCl_2_ in a Teflon-sealed, screw-capped 20-ml GC glass vial were performed. The volatile products were absorbed using a DVB/CAR/PDMS fiber in the headspace of the vial, which was incubated at 30°C for 1 hour. The fiber was inserted directly into the GC injector with a splitless injection. Volatile products were analyzed using GC–MS as described above. Heat-inactivated PdTPS proteins served as negative controls and were used under the same conditions.

For comparison of enzyme activities of PdTPS1 and PdTPS4, enzyme assays were conducted with 20 μg purified protein at each substrate concentration (20–160 μM GPP). The reaction was performed at 30°C for 20 minutes with the same reaction buffer as mentioned above. The content of product was quantitatively calculated from the calibration curve of linalool. Activities are expressed as nmol of product(s) formed/mg of lysate protein/hour.

### *In vivo* characterization of PdTPS1 and PdTPS4

The complete *PdTPS* ORFs were cloned into the *pSN1301* binary vector digested by BamHI using the 2 × Seamless Cloning Mix (Biomed). The constructed vectors were confirmed by DNA sequencing and then transformed into competent cells of *Agrobacterium tumefaciens* (strain GV3101). The leaf disk method was employed to transform 4-week-old *N. tabacum*. The *Agrobacterium* strain harboring empty vector was transformed as a negative control. The identified *T*_1_ generation of transgenic and negative control tobacco plants were grown in a greenhouse (25°C, 16 hours light/8 hours darkness). Volatiles from transgenic and control tobacco plants were extracted with a 50/30-μm DVB/CAR/PDMS fiber (Supelco, USA) for 3 hours at room temperature and analyzed as described above, but the injection mode was changed to splitless.

### Accession numbers

Sequence data from this article were deposited in GenBank under the following accession numbers: OM316806, PdTPS1; OM316807, PdTPS2; OM316808, PdTPS3; OM316809, PdTPS4; OM316810, PdTPS5.

## Supplementary Material

Web_Material_uhac253

## Data Availability

All the sequence read data are deposited in the NCBI Sequence Read Archive (SRA) under accession number PRJNA827976.
