# Supplementary material for: Biogenesis of flavor-related linalool is diverged and genetically conserved in tree peony (Paeonia × suffruticosa)
Source: Hortic Res. 2022 Nov 7;10(2):uhac253. doi: 10.1093/hr/uhac253 (PMC9896599; doi:10.1093/hr/uhac253)
Supplement: Web_Material_uhac253 [file web_material_uhac253.zip › Supplemental Files.docx]

## Supporting Information

Article title: Biogenesis of flavor-related linalool is diverged and genetically conserved in tree peony (*Paeonia* × *suffruticosa*)

Authors: Shanshan Li^1,2,3,†^, Ling Zhang^1,2,3,†^, Miao Sun^1,4^, Mengwen Lv^1,4^, Yong Yang^1,2^, Wenzhong Xu^1,2,3, ^[[1]](#footnote-0)^*^ and Liangsheng Wang^1,2,3, ^[[2]](#footnote-1)^*^

The following Supporting Information is available for this article:


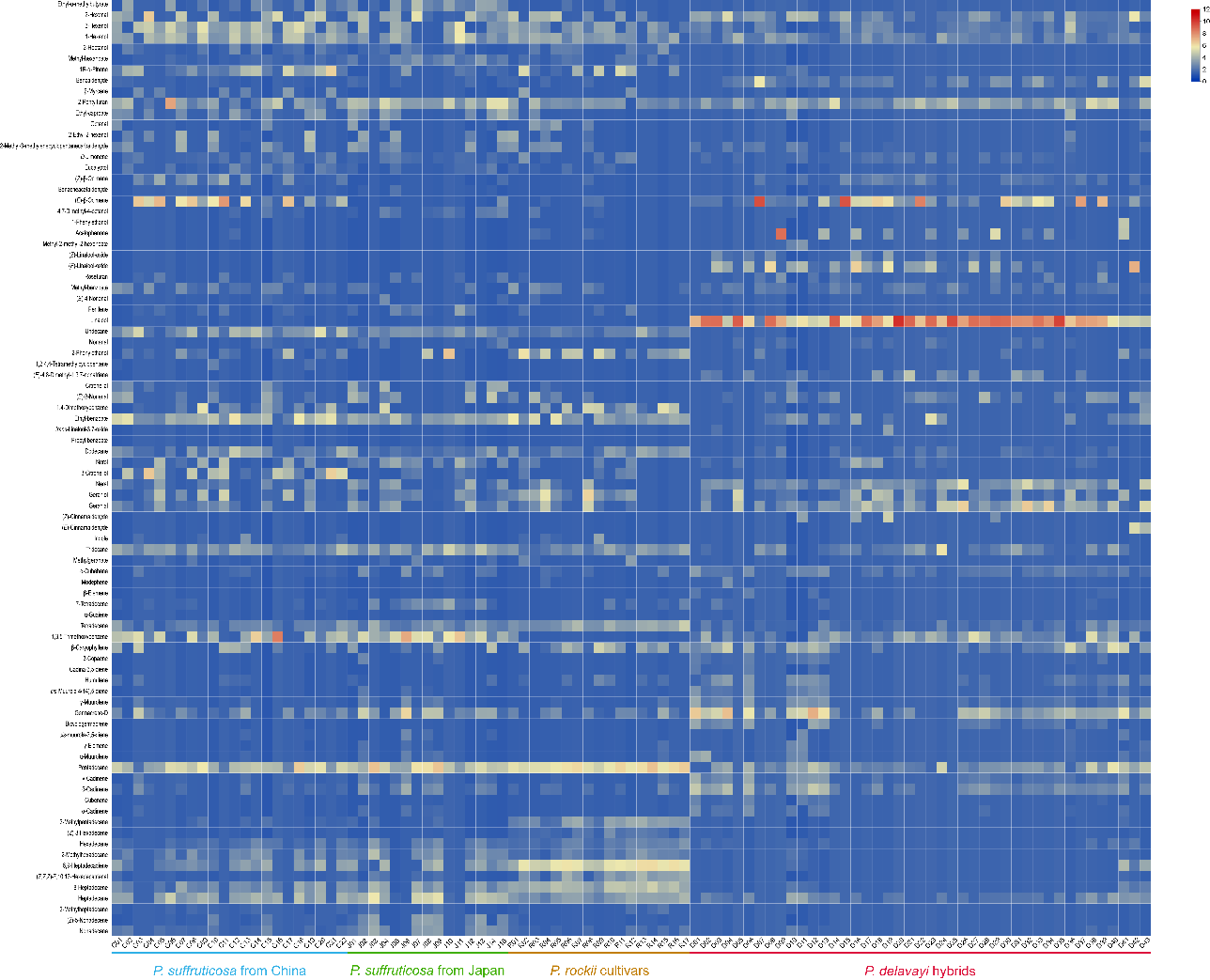


**Fig. S1 Composition and contents of volatile compounds released from different tree peony cultivars**

Cultivars were divided into four groups (*P.* × *suffruticosa* from China, *P.* × *suffruticosa* from Japan, *P. rockii* cultivars, and *P. delavayi* hybrids) depending on genetic background.

**
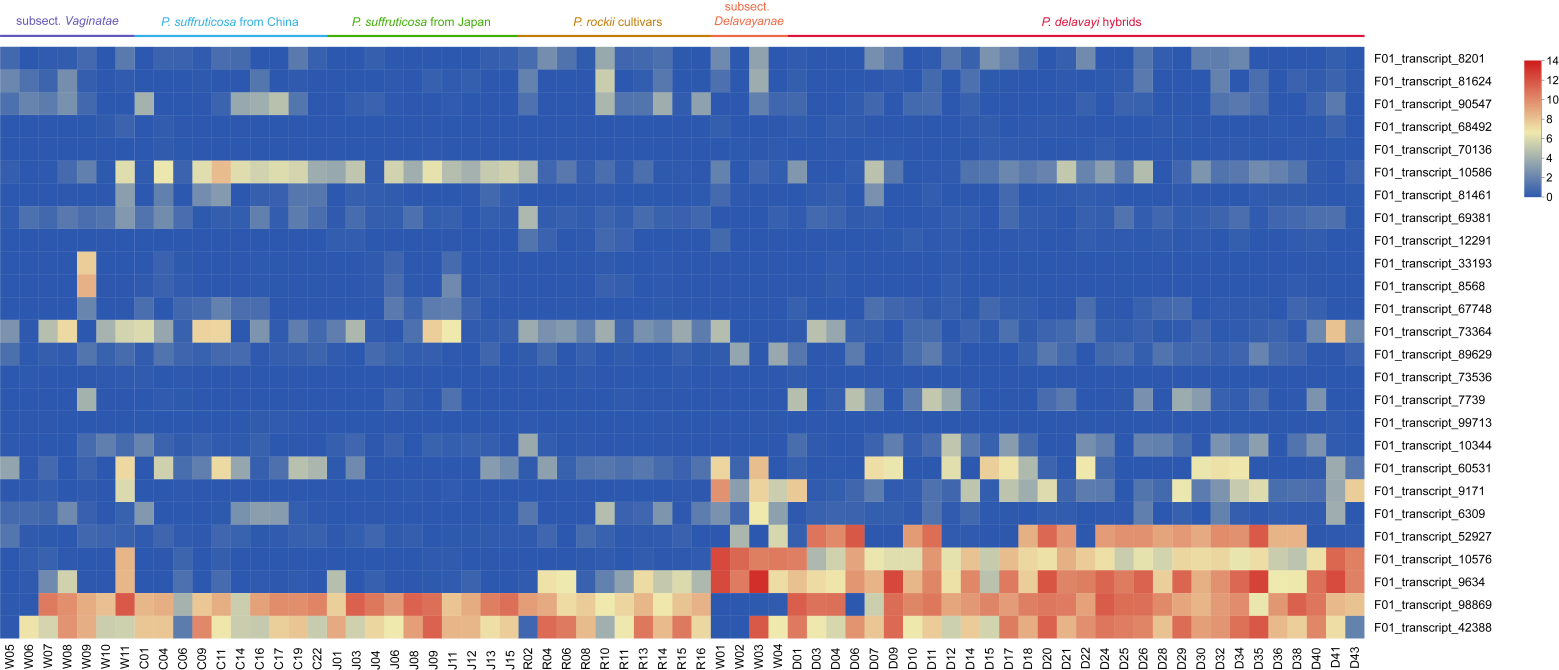
**

**Fig. S2** Distinct expression patterns of terpene synthases in tree peony


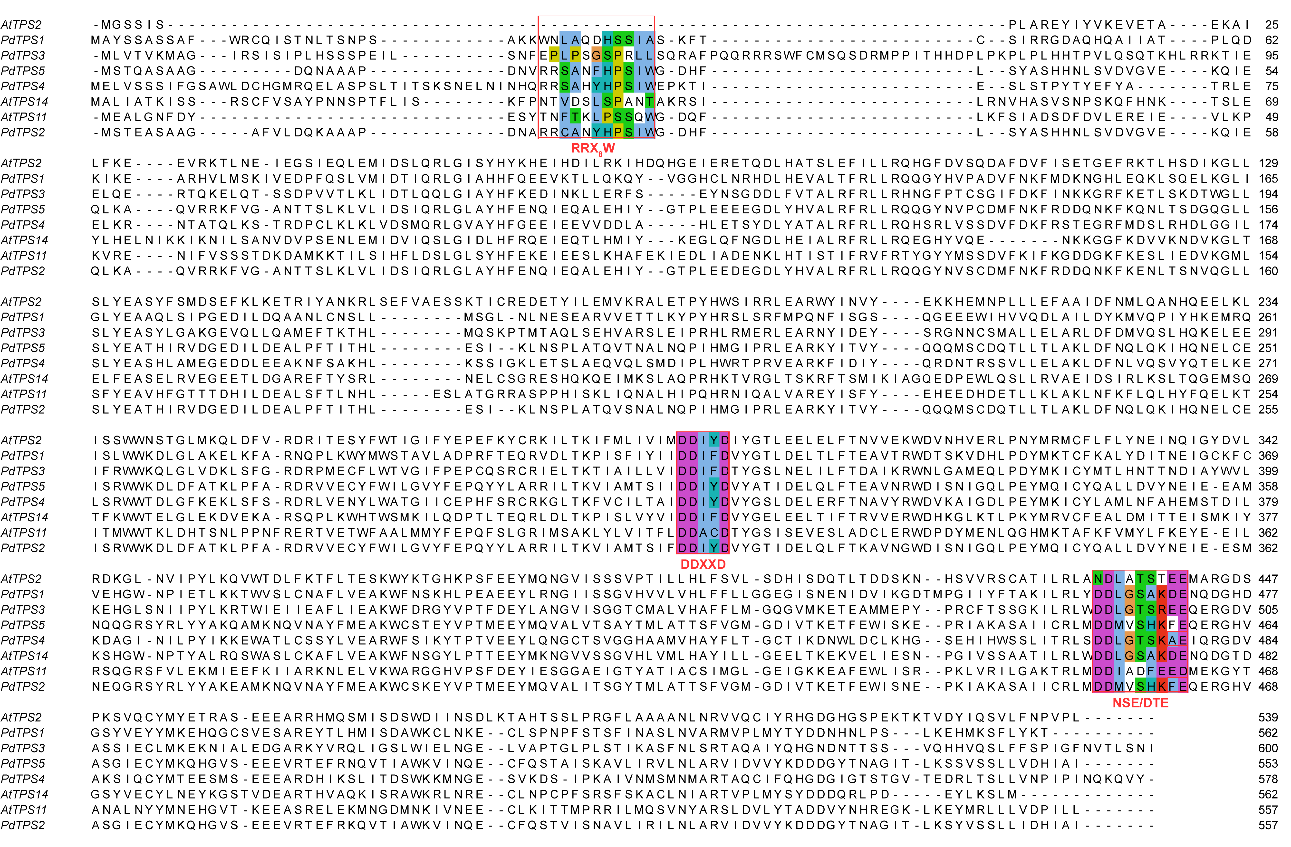


**Fig. S3 Sequence alignments of TPS proteins from tree peony and *Arabidopsis thaliana***

The sequences of the proteins encoded by PdTPS1 to PdTPS5 were aligned with sequences of the multi-product sesquiterpene synthase AtTPS11, trehalose-6-phosphate synthase AtTPS2, and linalool synthase AtTPS14. The conserved motifs RR(X)_8_W, DDXXD and NSE/DTE are highlighted with colored backgrounds.


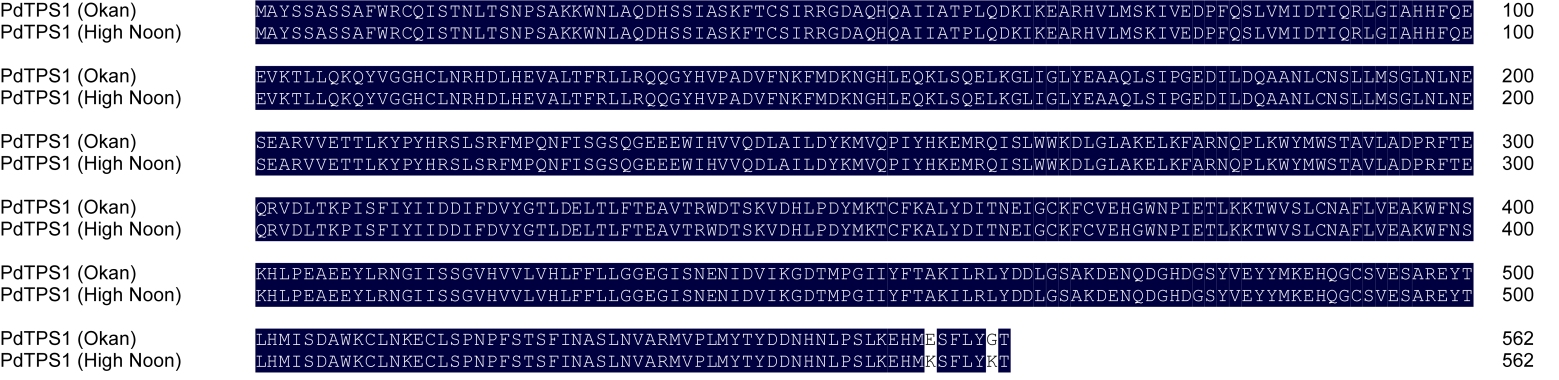


**Fig. S4** Amino acid sequence alignment of PdTPS1 proteins from different tree peony cultivars Multiple alignment analysis indicated that PdTPS1s from four tree peony cultivars were highly homologous to each other with identity value of 99.64%. The sequences of PdTPS1 from ‘High Noon’, ‘Narcissus’, ‘Bai He Zhan Chi’ and ‘Tian Xiang’ were identical. Amino acids conserved in all three sequences are shaded black.

**
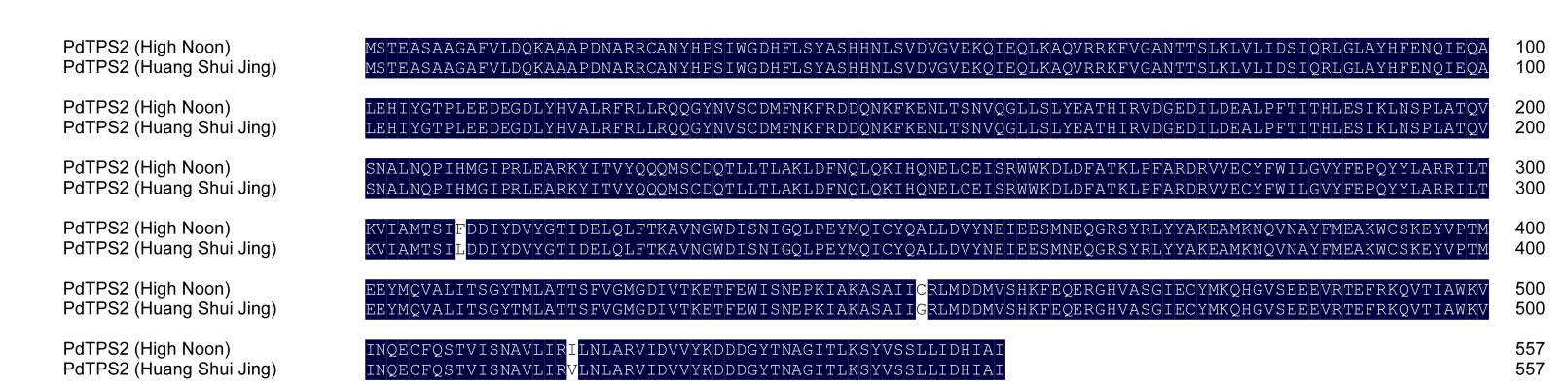
**

**Fig. S5** Amino acid sequence alignment of PdTPS2 proteins from different tree peony cultivars Multiple alignment analysis indicated that PdTPS2s from four tree peony cultivars were highly homologous to each other with identity value of 99.46%. The sequences of PdTPS2 from ‘High Noon’, ‘Okan’ and ‘Mei Xiang Hong’ were identical. Amino acids conserved in all three sequences are shaded black.

**
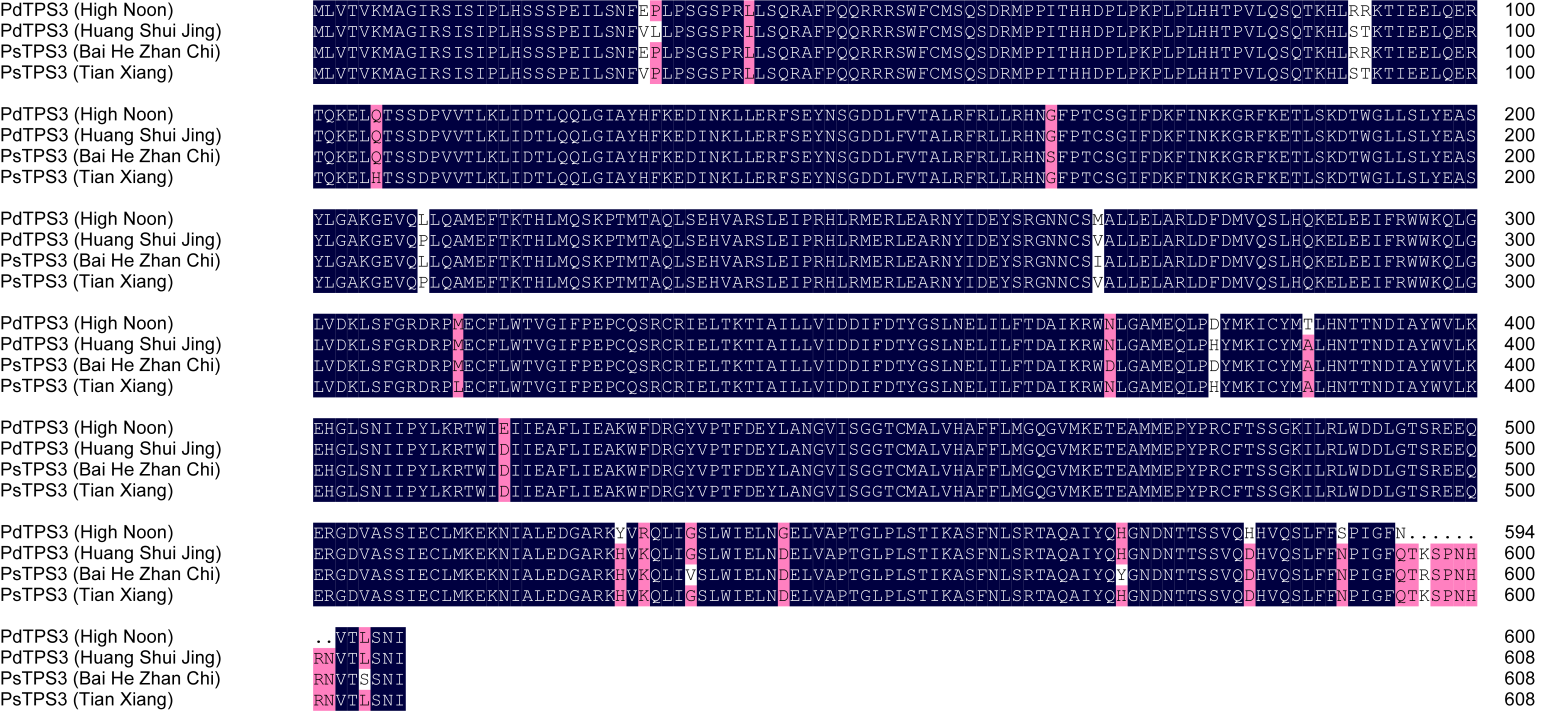
**

**Fig. S6** Amino acid sequence alignment of PdTPS3 proteins from different tree peony cultivars Multiple alignment analysis indicated that PdTPS3s from five tree peony cultivars were highly homologous to each other with identity value of 98.44%. The sequences of PdTPS3 from ‘High Noon’ and ‘Okan’ were identical. Amino acids conserved in all three sequences are shaded black, and those conserved in only three sequences are shaded hotpink.

**
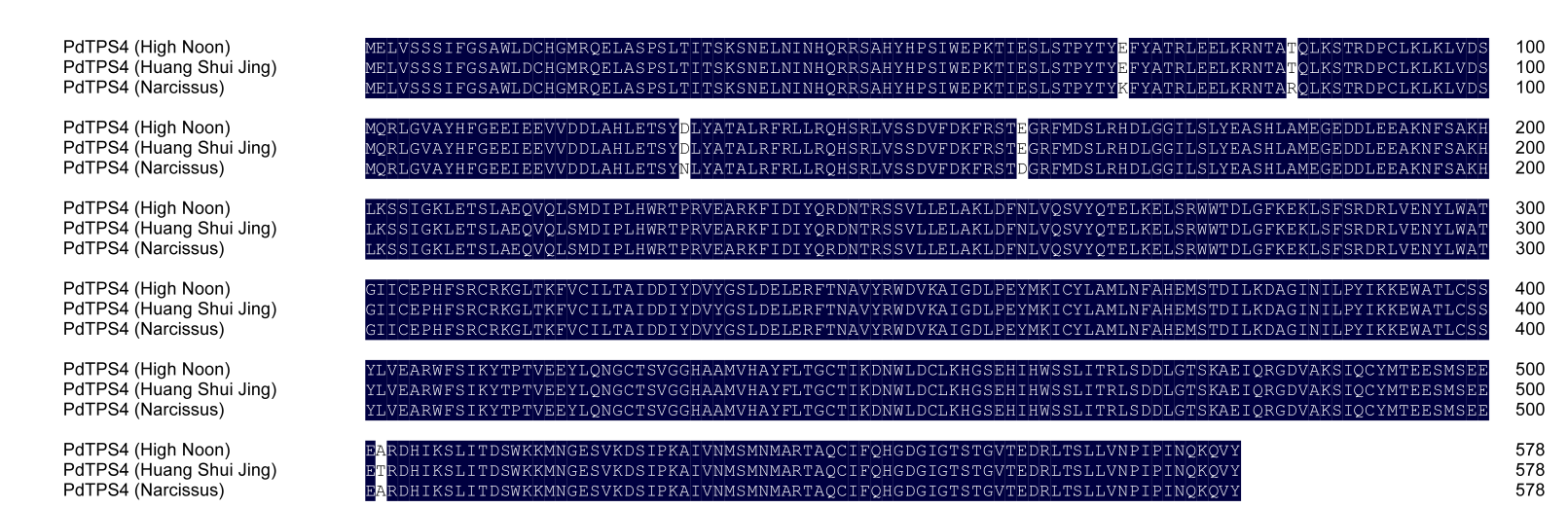
**

**Fig. S7** Amino acid sequence alignment of PdTPS4 proteins from different tree peony cultivars Multiple alignment analysis indicated that PdTPS4s from five tree peony cultivars were highly homologous to each other with identity value of 99.71%. The sequences of PdTPS4 from ‘High Noon’, ‘Okan’, ‘Bai He Zhan Chi’ and ‘Tian Xiang’ were identical. Amino acids conserved in all three sequences are shaded black.

**
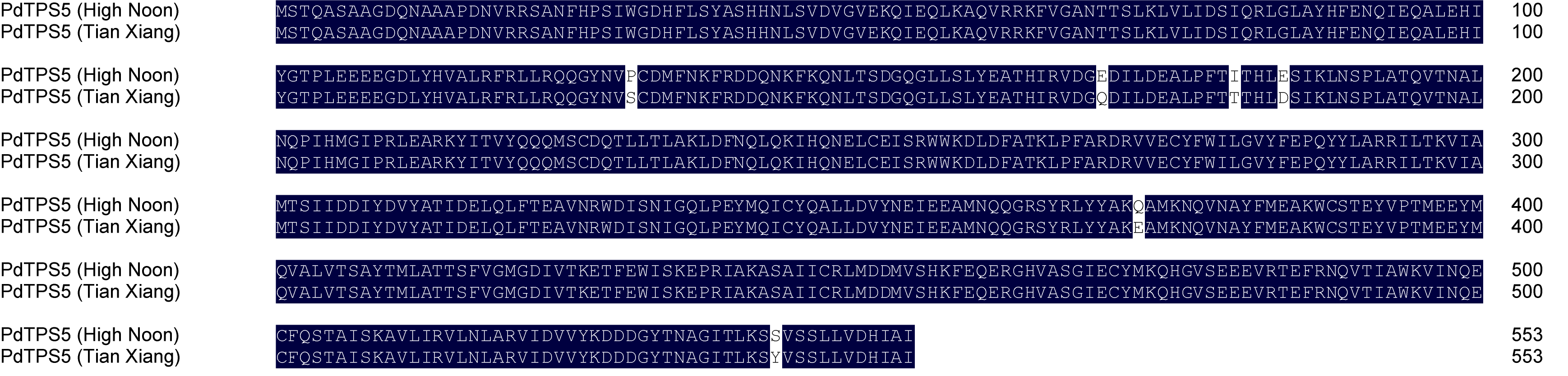
**

**Fig. S8** Amino acid sequence alignment of PdTPS5 proteins from different tree peony cultivars Multiple alignment analysis indicated that PdTPS4s from three tree peony cultivars were highly homologous to each other with identity value of 98.92%. The sequences of PdTPS5 from ‘High Noon’ and ‘Bai He Zhan Chi’ were identical. Amino acids conserved in all three sequences are shaded black.

**
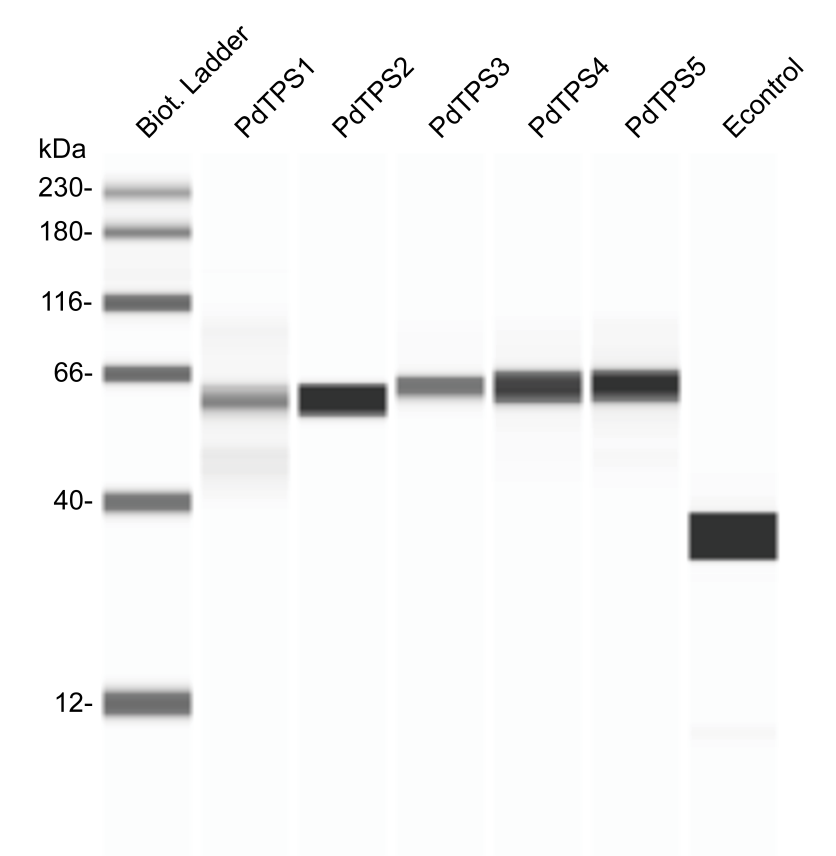
**

**Fig. S9** Western blot validation of the purified recombinant PdTPSs

**Table S1** Materials sampled in this study, including eight wild species of sect. *Moutan* and 97 cultivars

| **Category** | **Species / Cultivars** | **Country** | **Symbols** | **Category** | **Cultivars** | **Country** | **Symbols** | **Category** | **Cultivars** | **Country** | **Symbols** |
| --- | --- | --- | --- | --- | --- | --- | --- | --- | --- | --- | --- |
| Wild species | *Paeonia delavayi* | China | W01 | *P. × suffruticosa* from Japan | Shimanishiki | Japan | J05 | *P. delavayi* hybrids | Okan | Japan | D10 |
|  | *Paeonia delavayi* | China | W02 |  | Hyuhga | Japan | J06 |  | High Noon | USA | D11 |
|  | *Paeonia delavayi* | China | W03 |  | Kamatanishiki | Japan | J07 |  | Renown | USA | D12 |
|  | *Paeonia ludlowii* | China | W04 |  | Yachiyotsubaki | Japan | J08 |  | C22 | Australia | D13 |
|  | *Paeonia roundiloba* | China | W05 |  | Kao | Japan | J09 |  | C54 | Australia | D14 |
|  | *Paeonia decomposita* | China | W06 |  | Jitsugetsunishiki | Japan | J10 |  | Chinese Dragon | USA | D15 |
|  | *Paeonia rockii* subsp. *rockii* | China | W07 |  | Taiyo | Japan | J11 |  | CF1 | China | D16 |
|  | *Paeonia rockii* subsp. *atava* | China | W08 |  | Shimadaijin | Japan | J12 |  | C8 | Australia | D17 |
|  | *Paeonia qiui* | China | W09 |  | Shinshichifukujin | Japan | J13 |  | C18 | Australia | D18 |
|  | *Paeonia jishanensis* | China | W10 |  | Taishonohikari | Japan | J14 |  | CF2 | China | D19 |
|  | *Paeonia ostii* | China | W11 |  | Gosyozakura | Japan | J15 |  | C43 | Australia | D20 |
| *P. × suffruticosa* from China | Hu Lan | China | C01 | *P. rockii* cultivars | Ju Yuan Shao Nü | China | R01 |  | C33 | Australia | D21 |
|  | Gui Fei Cha Cui | China | C02 |  | Wan Xiang Yu | China | R02 |  | C6 | Australia | D22 |
|  | Xing Hua Chun Yu | China | C03 |  | Mu Ai | China | R03 |  | C1 | Australia | D23 |
|  | Bai He Zhan Chi | China | C04 |  | Zi Ban JJ-3 | China | R04 |  | C51 | Australia | D24 |
|  | Jing Yü | China | C05 |  | Qing Hai Hu Yin Bo | China | R05 |  | C37 | Australia | D25 |
|  | Dou Lü | China | C06 |  | Ri Yue Tong Hui | China | R06 |  | C2 | Australia | D26 |
|  | Gu Ban Tong Chun | China | C07 |  | Jing Yun Xiang | China | R07 |  | C7 | Australia | D27 |
|  | Hu Die Bao Chun | China | C08 |  | Zi Ban JJ-1 | China | R08 |  | Anna Marie | USA | D28 |
|  | Yao Huang | China | C09 |  | Wen Xian Fen | China | R09 |  | C12 | Australia | D29 |
|  | Liu Li Guan Zhu | China | C10 |  | Hei Tian E | China | R10 |  | C55 | Australia | D30 |
|  | Tian Xiang | China | C11 |  | Gu Cheng Xiang Hui | China | R11 |  | CX1 | Australia | D31 |
|  | Huo Lian Jin Dan | China | C12 |  | Jing Cheng Zi | China | R12 |  | C62 | Australia | D32 |
|  | Chu E Huang | China | C13 |  | Mo Guan Yin Xian | China | R13 |  | C24 | Australia | D33 |
|  | Cai Hui | China | C14 |  | Mei Gui Sa Jin | China | R14 |  | Savage Splendor | USA | D34 |
|  | Chun Hong Jiao Yan | China | C15 |  | Zong Ban Bai | China | R15 |  | C50 | Australia | D35 |
|  | Zhao Fen | China | C16 |  | Xue Hai Bing Xin | China | R16 |  | Leda | USA | D36 |
|  | Yin Hong Qiao Dui | China | C17 |  | Huang He Lou | China | R17 |  | C11 | Australia | D37 |
|  | Shou An Hong | China | C18 | *P. delavayi* hybrids | Narcissus | USA | D01 |  | Zephyrus | USA | D38 |
|  | Cao Zhou Hong | China | C19 |  | C17 | Australia | D02 |  | C26 | Australia | D39 |
|  | Hu Hong | China | C20 |  | Souvenir de Maxime Cornu | France | D03 |  | CX6 | Australia | D40 |
|  | Guan Shi Mo Yu | China | C21 |  | Chromatella | France | D04 |  | Huang Shui Jing | China | D41 |
|  | Xiang Yu | China | C22 |  | C36 | Australia | D05 |  | Zi Yan Gui Chao | China | D42 |
| *P. × suffruticosa* from Japan | Godaishu | Japan | J01 |  | Golden Isles | USA | D06 |  | Mei Xiang Hong | China | D43 |
|  | Muregarasu | Japan | J02 |  | Hephestos | USA | D07 |  |  |  |  |
|  | Hakuojishi | Japan | J03 |  | C30 | Australia | D08 |  |  |  |  |
|  | Sihimanechojuraku | Japan | J04 |  | Black Panther | USA | D09 |  |  |  |  |

**Table S2** Composition and average contents of volatiles emitted from flowers of wild tree peony species and cultivars (ng/g/h)

| **Compounds** | **RI** | **subsect. Vaginatae** | **subsect. Delavayanae** | ***P.* × suffruticosa from China** | ***P.* × suffruticosa from Japan** | ***P. rockii* cultivars** | ***P. delavayi* hybrids** |
| --- | --- | --- | --- | --- | --- | --- | --- |
| Ethyl-α-methylbutyrate | - | - | - | 2.21 | 5.90 | 0.20 | 0.04 |
| 2-Hexenal | - | 6.39 | 1.66 | 15.04 | 19.07 | 7.01 | 3.78 |
| 3-Hexenol | - | 0.52 | 0.04 | 12.81 | 4.57 | 6.10 | 0.53 |
| 1-Hexanol | - | 1.05 | 0.68 | 16.83 | 10.06 | 8.36 | 1.28 |
| 2-Heptanol | 901 | - | - | 0.49 | 0.35 | 1.32 | - |
| Methyl-hexanoate | 918 | - | - | 0.29 | 2.59 | 0.58 | - |
| 1R-α-Pinene | 938 | 0.25 | 1.11 | 8.15 | 0.53 | 13.29 | 0.12 |
| Benzaldehyde | 952 | 0.04 | 0.14 | 0.10 | 0.33 | 0.06 | 1.00 |
| β-Myrcene | 981 | - | - | 0.70 | - | 1.17 | - |
| 2-Pentylfuran | 987 | 1.22 | 0.25 | 12.97 | 60.69 | 5.94 | 3.28 |
| Ethyl-caproate | 996 | - | - | 2.99 | 8.16 | 0.59 | 0.46 |
| Octanal | 999 | 1.40 | - | 1.68 | 2.67 | 1.31 | 0.02 |
| 2-Ethyl-2-hexenal | 1003 | - | - | 2.70 | 3.41 | 1.15 | 0.03 |
| 2-Methyl-3-methylenecyclopentanecarbaldehyde | 1013 | 0.58 | 0.55 | 5.15 | 5.83 | 1.20 | 0.29 |
| D-Limonene | 1023 | 0.07 | 0.10 | 0.52 | 0.78 | 0.93 | 0.18 |
| Eucalyptol | 1025 | - | - | 0.97 | 0.47 | 0.19 | 0.00 |
| (Z)-β-Ocimene | 1034 | 0.10 | 0.20 | 1.85 | 0.10 | 0.03 | 0.28 |
| Benzeneacetaldehyde | 1038 | - | 0.22 | 0.02 | 0.15 | - | 0.01 |
| (E)-β-Ocimene | 1044 | 0.43 | 3.89 | 51.69 | 0.64 | 0.12 | 5.43 |
| 4,7-Dimethyl-4-octanol | 1053 | - | - | 0.36 | 1.24 | 0.15 | 0.02 |
| 1-Phenylethanol | 1055 | - | - | - | - | - | 0.26 |
| Acetophenone | 1060 | - | 16.92 | 0.02 | - | 0.12 | 0.95 |
| Methyl-2-methyl-2-hexenoate | 1066 | - | - | - | - | - | 0.35 |
| (Z)-Linalool-oxide | 1067 | - | 4.15 | - | - | - | 0.32 |
| (E)-Linalool-oxide | 1083 | - | 13.83 | - | - | - | 4.59 |
| Rosefuran | 1088 | 0.01 | 0.05 | 0.16 | 0.47 | 0.02 | 0.09 |
| Methyl-benzoate | 1089 | 0.51 | 0.04 | 2.10 | 0.63 | 1.33 | 0.26 |
| (E)-4-Nonenal | 1094 | - | - | 0.13 | 0.69 | 0.07 | - |
| Perillene | 1095 | - | - | 0.12 | 0.83 | 0.42 | - |
| Linalool | 1097 | - | 16.12 | - | - | - | 41.78 |
| Undecane | 1096 | 0.09 | - | 9.55 | 4.04 | 3.28 | - |
| Nonanal | 1101 | 0.01 | - | 0.19 | 0.73 | - | 0.09 |
| 2-Phenylethanol | 1107 | 3.67 | - | 1.19 | 2.59 | 19.68 | 0.12 |
| 1,2,4,4-Tetramethylcyclopentene | 1110 | - | - | 0.18 | - | - | - |
| (E)-4,8-Dimethyl-1,3,7-nonatriene | 1113 | - | - | - | - | - | 0.59 |
| Citronellal | 1149 | 0.21 | - | 2.65 | 12.43 | 0.03 | 0.22 |
| (E)-2-Nonenal | 1155 | 0.90 | - | 8.09 | 12.66 | 2.29 | 0.77 |
| 1,4-Dimethoxybenzene | 1159 | 13.74 | - | 6.07 | 21.26 | 21.37 | 0.06 |
| Ethyl benzoate | 1166 | 0.60 | 0.93 | 41.09 | 16.33 | 26.80 | 1.09 |
| trans-Linalool-3,7-oxide | 1169 | 0.01 | 1.04 | - | - | - | 0.09 |
| Propyl-benzoate | 1190 | 0.07 | - | - | - | - | - |
| Dodecane | 1196 | 0.13 | - | 5.04 | 4.04 | 4.80 | 0.07 |
| Nerol | 1223 | 0.06 | - | 1.95 | 4.37 | 0.83 | 0.24 |
| β-Citronellol | 1225 | 3.96 | - | 12.94 | 0.25 | 0.62 | - |
| Neral | 1236 | 0.61 | - | 1.95 | 7.80 | 0.93 | 1.76 |
| Geraniol | 1250 | 2.92 | - | 2.91 | 2.20 | 7.41 | 1.04 |
| Geranial | 1266 | 1.05 | - | 2.76 | 9.46 | 1.86 | 2.57 |
| (Z)-Cinnamaldehyde | 1268 | 0.10 | 0.76 | - | - | - | 0.71 |
| (E)-Cinnamaldehyde | 1269 | - | - | - | - | - | 0.35 |
| Indole | 1287 | 0.14 | - | 0.33 | - | 0.67 | - |
| Tridecane | 1296 | 0.39 | - | 6.49 | 14.47 | 5.95 | 0.53 |
| Methylgeranate | 1322 | - | - | 0.07 | 0.28 | 1.28 | - |
| α-Cubebene | 1345 | - | 0.22 | 0.13 | 0.94 | 0.29 | 0.65 |
| Modephene | 1372 | - | - | - | - | 0.02 | 0.01 |
| β-Elemene | 1388 | - | - | - | - | - | 0.08 |
| 7-Tetradecene | 1389 | 0.18 | - | 0.07 | 1.71 | 0.48 | 0.03 |
| α-Guaiene | 1399 | - | - | - | - | - | - |
| Tetradecane | 1405 | 0.28 | - | 3.10 | 5.01 | 13.06 | 0.24 |
| 1,3,5-Trimethoxybenzene | 1407 | 1.55 | - | 22.39 | 41.66 | 0.33 | 1.12 |
| β-Caryophyllene | 1413 | 0.41 | 0.74 | 3.20 | 3.92 | 11.98 | 3.07 |
| β-Copaene | 1423 | - | 0.18 | - | 0.24 | - | 0.21 |
| Cadina-3,5-diene | 1441 | - | - | - | - | - | 0.07 |
| Humulene | 1448 | 0.04 | 0.30 | 0.16 | 0.16 | 0.52 | 0.83 |
| cis-Muurola-4(14),5-diene | 1458 | - | - | - | 0.16 | - | 0.44 |
| γ-Muurolene | 1472 | - | 0.12 | 0.16 | 0.51 | 0.14 | 1.02 |
| Germacrene-D | 1476 | 0.17 | 1.22 | 2.54 | 10.50 | 2.68 | 12.36 |
| Bicyclogermacrene | 1490 | - | 0.09 | - | - | - | 0.57 |
| cis-muurola-3,5-diene | 1494 | - | 0.05 | 0.03 | 0.17 | - | 0.17 |
| γ-Elemene | 1496 | - | - | - | 0.12 | 0.08 | 0.33 |
| α-Muurolene | 1500 | - | 0.07 | 0.03 | 0.25 | 0.07 | 0.42 |
| Pentadecane | 1507 | 8.89 | - | 39.27 | 85.55 | 181.39 | 2.82 |
| γ-Cadinene | 1509 | - | 0.10 | 0.17 | 0.69 | 0.12 | 1.10 |
| δ-Cadinene | 1519 | 0.06 | 0.31 | 0.39 | 1.82 | 0.53 | 2.32 |
| Cubenene | 1527 | - | - | - | - | - | 0.19 |
| α-Cadinene | 1533 | - | 0.02 | 0.02 | 0.15 | - | 0.30 |
| 3-Methylpentadecane | 1538 | 0.18 | - | 0.35 | 0.20 | 8.59 | 0.01 |
| (Z)-8-Hexadecene | 1575 | - | - | 0.02 | - | 1.15 | - |
| Hexadecane | 1596 | 0.12 | - | 0.85 | 1.85 | 2.10 | 0.10 |
| 2-Methylhexadecane | 1660 | 0.14 | - | 2.48 | 4.03 | 2.27 | 0.09 |
| 6,9-Heptadecadiene | 1666 | 2.36 | - | 10.36 | 12.36 | 134.24 | 0.35 |
| (Z,Z,Z)-7,10,13-Hexadecatrienal | 1680 | - | - | 0.34 | 0.45 | 8.29 | 0.01 |
| 8-Heptadecene | 1685 | 2.64 | - | 5.17 | 8.05 | 26.66 | 0.18 |
| Heptadecane | 1696 | 2.90 | - | 9.78 | 28.26 | 9.59 | 1.73 |
| 3-Methylheptadecane | 1767 | 0.03 | - | 0.19 | 0.25 | 0.26 | 0.01 |
| (Z)-5-Nonadecene | 1785 | - | - | 0.62 | 3.57 | 0.03 | - |
| Nonadecane | 1813 | 0.24 | - | 1.48 | 4.65 | 0.47 | 0.14 |

**Table S3** Information of *PdTPS* genes isolated from ‘High Noon’

| **Candidate transcripts** | **PdTPS genes** | **GenBank** | **Protein sequence length (aa)** | **Top BLAST match** | **Homology (%)** | **Function prediction** |
| --- | --- | --- | --- | --- | --- | --- |
| F01_transcript_52927 | PdTPS1 | OM316806 | 562 residues | JQ062931.1  (3S)-linalool/(E)-nerolidol synthase  [Vitis vinifera cultivar Riesling] | 75 | Monoterpene synthase |
| F01_transcript_9634 | PdTPS2 | OM316807 | 557 residues | KU187412.1  terpenoid synthase mRNA  [Paeonia lactiflora] | 96 | Sesquiterpene synthase |
| F01_transcript_98869 | PdTPS3 | OM316808 | 600 residues | MN639696.1  geraniol synthase  [Rosa x damascena] | 80 | Monoterpene synthase |
| F01_transcript_10576 | PdTPS4 | OM316809 | 578 residues | XM_002275070.3  terpene synthase 9  [Vitis vinifera] | 74 | Monoterpene synthase |
| F01_transcript_42388 | PdTPS5 | OM316810 | 553 residues | KU187412.1  terpenoid synthase mRNA  [Paeonia lactiflora] | 96 | Sesquiterpene synthase |

**Table S4** TPS proteins from other plant species used in phylogenetic analysis

| **Species** | **Protein** | **Protein ID in NCBI** | **Note** |
| --- | --- | --- | --- |
| *Abies grandis* | AgfEabis | O81086.1 | (*E*)-Alpha-bisabolene synthase |
| *Arabidopsis thaliana* | AtTPS2 | NP 193406.3 | (E)-Bate-ocimene/myrcene synthase |
|  | AtTPS11 | ABO09887.1 | (*E*)-Beta caryophyllene synthase |
|  | AtTPS14 | NP 001185286.1 | (±)-3S-Linalool synthase |
|  | AtTPS24 | NP 189209.2 | 1,8-Cineole synthase |
|  | AtKS | AAC39443.1 | Ent-kaurene synthase |
| *Cannabis sativa* | CsTPS2 | ABI21838.1 | (+)-Alpha-pinene synthase |
| *Cinnamomum tenuipilum* | CtGES | AJ457070.2 | Geraniol synthase |
| *Gossypium hirsutum* | GhCPS | AIY27525.1 | Ent-copalyl diphosphate synthase |
|  | GhTPS2 | AFQ23190.1 | Alpha-pinene synthase |
| *Hedychium coronarium* | HcTPS8 | AGY49283.1 | Linalool synthase |
|  | HcTPS7 | AHJ57305.1 | Sabinene synthase |
| *Lavandula angustifolia* | LaCARS | AGL98419.1 | Caryophyllene synthase |
|  | LaGERDS | AGL98420.1 | Ggermacrene D synthase |
|  | LaLINS | Q2XSC5.1 | Linalool synthase |
| *Medicago truncatula* | MtCPS | KEH21632.1 | Copalyl diphosphate synthase |
|  | MtTPS15 | XP 003621227.1 | Alpha-farnesene synthase |
|  | MtTPS23 | XP 003619707.1 | Nerolidol synthase |
| *Ocimum basilicum* | ObGES | AAR11765.1 | Geraniol synthase |
| *Oryza sativa* | OsGerD | XP_015629743.1 | (+)-Germacrene D synthase |
| *Pinus taeda* | PtAO | AAX07435.1 | Diterpene synthase |
| *Solanum lycopersicum* | SlTPS25 | NP_001308094.1 | (*E*)-Beta-ocimene synthase |
|  | SlTPS5 | NP_001233805.1 | Linalool synthase |
|  | SlTPS24 | NP_001307929.1 | Ent-kaurene synthase |
|  | SlTPS40 | NP 001234008.2 | Copalyl-diphosphate synthase |
|  | SlTPS8 | XP 004231365.1 | 1,8-Cineole synthase |
|  | SlTPS12 | NP_001234766.1 | Caryophyllene/alpha-humulene synthase |
| *Vitis vinifera* | VvGwbOci | ADR74205.1 | (*E*)-Beta-ocimene synthase |
|  | VvGwGerA | ADR66821.1 | germacrene A synthase |
|  | VvGwaBer | [ADR74195.2](https://www.ncbi.nlm.nih.gov/protein/315115210) | (E)-alpha-bergamotene synthase |
|  | VvGwgCad | ADR74199.1 | Gamma-cadinene synthase |
|  | VvGwGerD | AAS66357.1 | (-)-Germacrene D synthase |
|  | VvPNGer | ADR74218.1 | Geraniol synthase |
|  | VvPNaPIN | ADR74203.1 | (+)-Alpha-pinene synthase |
| *Zea mays* | ZmTPS6 | NP 001105674.1 | (*S*)-Beta-macrocarpene synthase |
|  | ZmTPS10 | NP 001105850.1 | (*E*)-Beta farnesene synthase |
|  | ZmTPS23 | ABY79213.1 | (*E*)-Beta caryophyllene synthase |

**Table S5** Enzymatic products catalyzed by PdTPS proteins

| **Enzymatic**  **products** | **PdTPS1** | | | |  | **PdTPS2** | | | |  | **PdTPS3** | | | |  | **PdTPS4** | | | |  | **PdTPS5** | | | |
| --- | --- | --- | --- | --- | --- | --- | --- | --- | --- | --- | --- | --- | --- | --- | --- | --- | --- | --- | --- | --- | --- | --- | --- | --- |
|  | **GPP** | **NPP** | ***E,E*-FPP** | ***Z,Z*-FPP** |  | **GPP** | **NPP** | ***E,E*-FPP** | ***Z,Z*-FPP** |  | **GPP** | **NPP** | ***E,E*-FPP** | ***Z,Z*-FPP** |  | **GPP** | **NPP** | ***E,E*-FPP** | ***Z,Z*-FPP** |  | **GPP** | **NPP** | ***E,E*-FPP** | ***Z,Z*-FPP** |
| **Monoterpene** |  |  |  |  |  |  |  |  |  |  |  |  |  |  |  |  |  |  |  |  |  |  |  |  |
| β-Myrcene | - | - | - | - |  | 61.7% | - | - | - |  | - | - | - | - |  | - | - | - | - |  | - | - | - | - |
| D-Limonene | - | - | - | - |  | 15.9% | 67.2% | - | - |  | - | - | - | - |  | - | - | - | - |  | 100% | 72.3% | - | - |
| Terpinolene | - | - | - | - |  | 7.8% | 28.1% | - | - |  | - | - | - | - |  | - | - | - | - |  | - | 27.7% | - | - |
| Linalool | 100% | - | - | - |  | - | - | - | - |  | - | - | - | - |  | 100% | - | - | - |  | - | - | - | - |
| Camphene | - | - | - | - |  | 2.7% | - | - | - |  | - | - | - | - |  | - | - | - | - |  | - | - | - | - |
| α-Terpineol | - | - | - | - |  | - | 4.8% | - | - |  | - | - | - | - |  | - | 100% | - | - |  | - | - | - | - |
| (-)-β-Pinene | - | - | - | - |  | 3.6% | - | - | - |  | - | - | - | - |  | - | - | - | - |  | - | - | - | - |
| Geraniol | - | - | - | - |  |  | - | - | - |  | 100% | - | - | - |  | - | - | - | - |  | - | - | - | - |
| β-Pinene | - | - | - | - |  | 8.3% | - | - | - |  | - | - | - | - |  | - | - | - | - |  | - | - | - | - |
| **Sesquiterpene** |  |  |  |  |  |  |  |  |  |  |  |  |  |  |  |  |  |  |  |  |  |  |  |  |
| γ-Elemene | - | - | - | - |  | - | - | 5.7% | - |  | - | - | - | - |  | - | - | - | - |  | - | - | - | - |
| cis-Calamenene | - | - | - | - |  | - | - | 1.6% | - |  | - | - | - | - |  | - | - | - | - |  | - | - | - | - |
| (-)-β-Elemene | - | - | - | - |  | - | - | 3.6% | 18.7% |  | - | - | - | - |  | - | - | - | - |  | - | - | - | - |
| β-Caryophyllene | - | - | - | - |  | - | - | 7.4% | - |  | - | - | - | - |  | - | - | - | - |  | - | - | - | - |
| β-Copaene | - | - | - | - |  | - | - | 3.3% | - |  | - | - | - | - |  | - | - | - | - |  | - | - | - | - |
| Aromandendrene | - | - | - | - |  | - | - | 2.2% | - |  | - | - | - | - |  | - | - | - | - |  | - | - | - | - |
| Alloaromadendrene | - | - | - | - |  | - | - | 4.0% | - |  | - | - | - | - |  | - | - | - | - |  | - | - | - | - |
| (-)-β-Cadinene | - | - | - | - |  | - | - | 4.4% | - |  | - | - | - | - |  | - | - | - | - |  | - | - | - | - |
| γ-Muurolene | - | - | - | - |  | - | - | 5.2% | - |  | - | - | - | - |  | - | - | - | - |  | - | - | - | - |
| trans-Nuciferol | - | - | - | - |  | - | - | - | 6.1% |  | - | - | - | - |  | - | - | - | - |  | - | - | - | - |
| Germacrene D | - | - | - | - |  | - | - | 27.0% | - |  | - | - | - | - |  | - | - | - | - |  | - | - | 64.3% | - |
| Curcumene | - | - | - | - |  | - | - | - | 3.7% |  | - | - | - | - |  | - | - | - | - |  | - | - | - | - |
| Patchoulene | - | - | - | - |  | - | - | 14.7% | - |  | - | - | - | - |  | - | - | - | - |  | - | - | - | - |
| γ-Elemene | - | - | - | - |  | - | - | - | - |  | - | - | - | - |  | - | - | - | - |  | - | - | 35.7% | - |
| α-Muurolene | - | - | - | - |  | - | - | 2.9% | - |  | - | - | - | - |  | - | - | - | - |  | - | - | - | - |
| β-Bisabolene | - | - | - | - |  | - | - | - | 19.9% |  | - | - | - | - |  | - | - | - | - |  | - | - | - | 72.2% |
| γ-Cadinene | - | - | - | - |  | - | - | 6.4% | - |  | - | - | - | - |  | - | - | - | - |  | - | - | - | - |
| Longipinene | - | - | - | - |  | - | - | - | 27.5% |  | - | - | - | - |  | - | - | - | - |  | - | - | - | - |
| cis-γ-Bisabolene | - | - | - | - |  | - | - | - | - |  | - | - | - | - |  | - | - | - | - |  | - | - | - | 27.8% |
| δ-Cadinene | - | - | - | - |  | - | - | 9.7% | - |  | - | - | - | - |  | - | - | - | - |  | - | - | - | - |
| trans-γ-Bisabolene | - | - | - | - |  | - | - | - | 16.9% |  | - | - | - | - |  | - | - | - | - |  | - | - | - | - |
| α-Cadinene | - | - | - | - |  | - | - | 1.9% | - |  | - | - | - | - |  | - | - | - | - |  | - | - | - | - |
| trans-α-Bisabolene | - | - | - | - |  | - | - | - | 7.2% |  | - | - | - | - |  | - | - | - | - |  | - | - | - | 31.1% |

**Table S6** Enzyme activities of PdTPS1 and PdTPS4 at different substrate (GPP) concentrations

| GPP concentrations (μM) | PdTPS1 | PdTPS4 |
| --- | --- | --- |
| 20 | 0.40±0.06 | 0.49±0.03 |
| 40 | 0.76±0.08 | 0.90±0.06 |
| 80 | 1.60±0.08 | 1.43±0.16 |
| 120 | 2.38±0.15 | 2.54±0.17 |
| 160 | 4.36±0.22 | 4.65±0.27 |

Enzyme assays were conducted with 20 µg purified PdTPS1 and PdTPS4 protein at different substrate concentrations. Activities are expressed as nmol of linalool formed/μg of lysate protein/h (n=3).

**Table S7** The primer pairs used in the present study

|  |  | **Forward (5'-3')** | **Reverse (5'-3')** |
| --- | --- | --- | --- |
| Full-length gene amplification | *PdTPS1* | ATGGCCTATTCCAGTGCCTC | TTAAGTTTTATACAAGAATGATTTCATGTGCTCC |
|  | *PdTPS2* | ATGTCTACTGAAGCTTCCGC | TCATATTGCAATATGATCAATGAGTAATG |
|  | *PdTPS3* | ATGCTTGTTACAGTGAAAATGGCTGG | TCATATGTTTGACAAAGTTACATTAAAACCAATGGG |
|  | *PdTPS4* | ATGGAACTAGTCTCTTCCTCCATTTTC | TCAATAGACTTGCTTTTGATTAATCGGAATAG |
|  | *PdTPS5* | ATGTCTACTCAAGCTTCCGCTGC | TCATATTGCAAAATGATCAACGAGTAATG |
| Generation of constructs used in subcellular localization | *PdTPS1* | GGTCGACATTTAAATACTAGTATGGCCTATTCCAGTGC | CATGGTACCGGATCCACTAGTAGTTTTATACAAGAATGATTTC |
|  | *PdTPS2* | GGTCGACATTTAAATACTAGTATGTCTACTGAAGCT | CATGGTACCGGATCCACTAGTTATTGCAATATGATC |
|  | *PdTPS3* | GGTCGACATTTAAATACTAGTATGCTTGTTACAGTG | CATGGTACCGGATCCACTAGTTATGTTTGACAAAGT |
|  | *PdTPS4* | GGTCGACATTTAAATACTAGTATGGAACTAGTCTCT | CATGGTACCGGATCCACTAGTATAGACTTGCTTTTG |
|  | *PdTPS5* | GGTCGACATTTAAATACTAGTATGTCTACTCAAGCT | CATGGTACCGGATCCACTAGTTATTGCAATATGATC |
| Generation of constructs used in heterologous expression in tobacco | *PdTPS1* | CACGGGGGACTCTAGAGGATCCATGGCCTATTCCAGTGC | GAGCTCGGTACCCGGGGATCCTTAAGTTTTATACAAGAAT |
|  | *PdTPS2* | CACGGGGGACTCTAGAGGATCCATGTCTACTGAAGCT | GAGCTCGGTACCCGGGGATCCTCATATTGCAATATG |
|  | *PdTPS3* | CACGGGGGACTCTAGAGGATCCATGCTTGTTACAGTG | GAGCTCGGTACCCGGGGATCCTCATATGTTTGACAA |
|  | *PdTPS4* | CACGGGGGACTCTAGAGGATCCATGGAACTAGTCTCTT | GAGCTCGGTACCCGGGGATCCTCAATAGACTTGCTTT |
|  | *PdTPS5* | CACGGGGGACTCTAGAGGATCCATGTCTACTCAAGCT | GAGCTCGGTACCCGGGGATCCTCATATTGCAATATG |
| qRT-PCR | *PdTPS1* | CATGATCGACACCATCCAGC | AACACATCTGCAGGCACATG |
|  | *PdTPS2* | GCTTGTATGAGGCTACGCAC | TGCGAGTGTGAGGAGAGTTT |
|  | *PdTPS3* | GAACACGTTGCCAGGTCTTT | AGAAGCACTCCATTGGTCGA |
|  | *PdTPS4* | ATGGGTGTACATCGGTTGGT | TGAATCTCGGCCTTGGAAGT |
|  | *PdTPS5* | GCTTGTATGAGGCTACGCAC | TGCGAGTGTGAGGAGAGTTT |

Note: the base sequences with underlines indicate restriction enzyme sites.

1. *To whom correspondence may be addressed. Tel: +86 10 6283 6654. E-mail: wanglsh@ibcas.ac.cn; Tel: +86 10 6283 6654. E-mail: xuwzh@ibcas.ac.cn. [↑](#footnote-ref-0)
2. [↑](#footnote-ref-1)
